# Translation control during prolonged mTORC1 inhibition mediated by 4E-BP3

**DOI:** 10.1038/ncomms11776

**Published:** 2016-06-20

**Authors:** Yoshinori Tsukumo, Tommy Alain, Bruno D. Fonseca, Robert Nadon, Nahum Sonenberg

**Affiliations:** 1 Department of Biochemistry and Goodman Cancer Research Centre, McGill University, Cancer Pavilion 1160 Pine Avenue West, Montreal, Quebec, Canada H3A 1A3; 2 Department of Biochemistry, Microbiology and Immunology, Children's Hospital of Eastern Ontario Research Institute, University of Ottawa, Ottawa, Ontario, Canada K1H 8L1; 3 McGill University and Genome Quebec Innovation Centre, Department of Human Genetics, McGill University, Montreal, Quebec, Canada H3A 1A5

## Abstract

Targeting mTORC1 is a highly promising strategy in cancer therapy. Suppression of mTORC1 activity leads to rapid dephosphorylation of eIF4E-binding proteins (4E-BP1–3) and subsequent inhibition of mRNA translation. However, how the different 4E-BPs affect translation during prolonged use of mTOR inhibitors is not known. Here we show that the expression of 4E-BP3, but not that of 4E-BP1 or 4E-BP2, is transcriptionally induced during prolonged mTORC1 inhibition *in vitro* and *in vivo*. Mechanistically, our data reveal that 4E-BP3 expression is controlled by the transcription factor TFE3 through a *cis*-regulatory element in the *EIF4EBP3* gene promoter. CRISPR/Cas9-mediated *EIF4EBP3* gene disruption in human cancer cells mitigated the inhibition of translation and proliferation caused by prolonged treatment with mTOR inhibitors. Our findings show that 4E-BP3 is an important effector of mTORC1 and a robust predictive biomarker of therapeutic response to prolonged treatment with mTOR-targeting drugs in cancer.

## Abstract (web summary)

The eIF4E-binding proteins (4E-BPs) are critical repressors of cap-dependent translation via mTOR, a pathway frequently hyperactivated in cancer. Here the authors show that 4E-BP3 specifically mediates the cap-dependent translation repression and antiproliferative effects of prolonged pharmacological mTOR inhibition.

## Graphical abstract

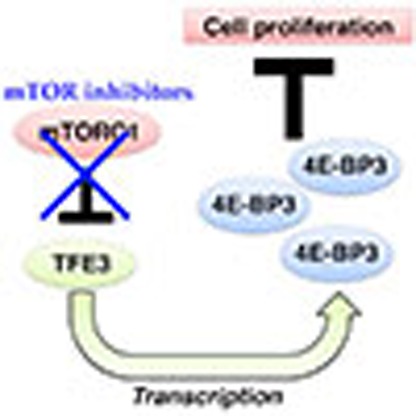

The mechanistic/mammalian target of rapamycin (mTOR) is a multifaceted serine/threonine kinase that has been implicated in a large number of physiological processes and pathological states including cancer^1^,^2^,^3^. mTOR forms two distinct complexes, mTOR complex 1 (mTORC1) and 2 (mTORC2), which differ in their composition, downstream targets, regulation and sensitivity to the mTOR allosteric inhibitor rapamycin^1^,^4^,^5^,^6^. mTORC1 stimulates translation by phosphorylating downstream targets including the eukaryotic translation initiation factor 4E (eIF4E)-binding proteins (4E-BPs) and ribosomal protein S6 kinases^7^,^8^. Under nutrient-rich conditions, hyperphosphorylation of 4E-BPs by mTORC1 releases 4E-BPs from (eIF4E), the messenger RNA 5′-cap-binding subunit of the eIF4F complex, and promotes the recruitment of a subset of mRNAs to the ribosomes^8^,^9^,^10^,^11^. Under poor nutrient conditions or pharmacological inhibition of mTORC1, 4E-BPs become hypophosphorylated and bind to eIF4E with high affinity, preventing eIF4F complex assembly and translation initiation^8^,^9^,^10^,^11^.

mTORC1 is frequently hyperactivated in a variety of cancers. Thus, there is considerable interest in developing therapeutic strategies that target aberrant mTORC1 activation in cancer^1^,^12^. Highly specific inhibitors of mTORC1, rapamycin and its analogues (rapalogs), are in the clinic for treatment of advanced renal cell carcinoma (RCC) and pancreatic neuroendocrine tumours (PNETs)^12^,^13^,^14^. Rapalogs, in general, exhibit modest anti-cancer efficacy, which is partly due to incomplete inhibition of the phosphorylation of 4E-BPs^15^,^16^. Newly developed mTOR inhibitors, which target the active site of mTOR (asTORi or TORKi; for example, PP242 and MLN0128) abolish the phosphorylation of 4E-BPs and exhibit improved anti-proliferative and anti-tumorigenic effects as compared with rapamycin^12^,^15^,^16^. Proliferation of 4E-BP1- and 2-depleted cells is resistant to pharmacological inhibition of mTOR, which can be explained by the sustained upregulation of the translation of a subset of eIF4E-sensitive mRNAs encoding pro-proliferative proteins (cyclin D3, cyclin E1 and vascular endothelial growth factor) and pro-invasive proteins (Y-box protein 1, vimentin and CD44)^17^,^18^,^19^. Thus, the mTORC1–4E-BP1 axis plays an important role in tumour development and drug response. The mTORC1–4E-BP1 axis has been also used as a surrogate marker to predict patient outcome in several cancers^20^,^21^,^22^,^23^.

In this study, we investigated the regulatory mechanism and molecular function of the third 4E-BP, 4E-BP3, which has not been well studied. It shares an eIF4E-binding site and the major phosphorylation sites with 4E-BP1 and 4E-BP2, but it appears to be weakly phosphorylated probably due to the lack of an amino-terminal RAIP motif, which effects insulin-stimulated phosphorylation^24^,^25^. Our study shows that 4E-BP3 is mainly regulated by transcriptional induction downstream of the mTORC1 pathway, in sharp contrast to 4E-BP1 and 2, which are controlled by phosphorylation^9^,^10^. We also show that 4E-BP3 induction is mediated by the MiTF (microphthalmia-associated transcription factor) family transcription factor TFE3, which is known to be activated during mTORC1 inhibition^26^,^27^,^28^. Ablation of 4E-BP3 in cancer cells reveals that it plays an important role in controlling translation of eIF4E-target mRNAs and cell proliferation. Our data demonstrate that under prolonged mTORC1 inhibition, 4E-BP3 becomes an important effector downstream of mTORC1 in a unique mechanism that differs from that of 4E-BP1 and 2.

## Results

### 4E-BP3 is transcriptionally induced during mTORC1 inhibition

**Figure 1:**
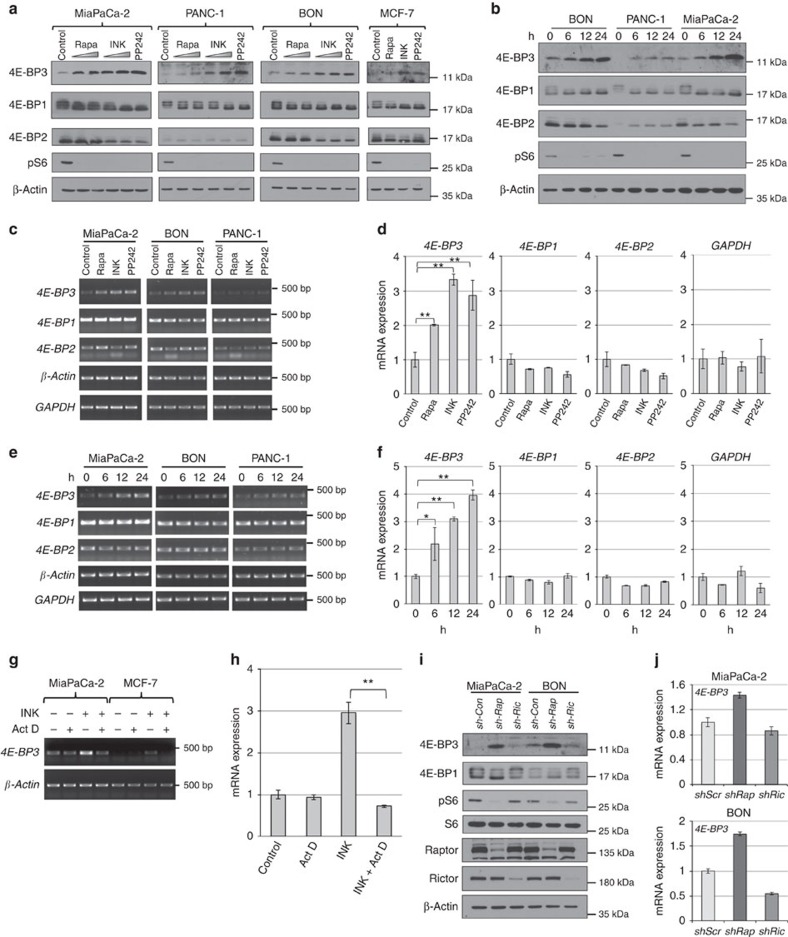
4E-BP3 is transcriptionally induced by mTORC1 inhibition. (**a**) MiaPaCa-2, PANC-1, BON and MCF-7 cells were treated with rapamycin (10 and 100 nM), INK1341 (30 and 100 nM) or PP242 (1 μM) for 24 h. Expression of the indicated proteins was measured by immunoblotting. The anti-4E-BP1 or -4E-BP2 antibody detects both hypo- and hyperphosphorylated forms of 4E-BPs. (**b**) MiaPaCa-2, BON and PANC-1 cells were treated with 100 nM of INK1341 for the indicated time. (**c**) MiaPaCa-2, BON and PANC-1 cells were treated with rapamycin (100 nM), INK1341 (100 nM) or PP242 (1 μM) for 24 h. mRNA expression was determined by RT–PCR analysis. (**d**) *4E-BPs* expression in MiaPaCa-2 cells was analysed by real-time PCR. Expression of *4E-BP*s was normalized against β-Actin. Error bars indicate±s.d. (*n*=3). **P<0.01. Statistical significance was determined using one-way analysis of variance (ANOVA). (**e**) Cells were treated with 100 nM of INK1341 for the indicated times. mRNA expression was determined by RT–PCR analysis. (**f**) *4E-BPs* expression in MiaPaCa-2 cells was analysed by real-time PCR. Expression of *4E-BP*s was normalized against β-Actin. Error bars indicate±s.d. (*n*=3). *P<0.05 and **P<0.01. Statistical significance was determined using one-way ANOVA. (**g**,**h**) MiaPaCa-2 and MCF-7 cells were treated with 100 nM of INK1341 in the presence or absence of actinomycin D (5 μg ml^−1^) for 18 h. mRNA level was determined by RT–PCR (**g**) or real-time PCR analysis for MiaPaCa-2 (**h**). Error bars indicate ±s.d. (*n*=3). **P<0.01. Statistical significance was determined using Student *t*-test. (**i**,**j**) MiaPaCa-2 and BON cells were transduced with scrambled (sh-Scr), raptor (sh-Rap) or rictor (sh-Ric) shRNA-lentivirus. Equal amount of protein was loaded on separate gels in parallel, and each protein expression was determined by immunoblotting. Arrow indicates nonspecific band (**i**). mRNA expression was determined by real-time PCR analysis (**j**). (**a**–**j**) A representative result of three independent experiments for MiaPaCa-2 and MCF-7 cells or two independent experiments for BON and PANC-1 cells is shown.

To study the function of 4E-BP3 downstream of mTORC1, we examined the effect of mTOR inhibitors (rapamycin, or asTORi, INK1341 and PP242) on the expression of 4E-BP3 in a panel of human cancer cell lines. Prolonged treatment of human pancreatic cancer cell lines MiaPaCa-2, PANC1 and BON, and the breast cancer cell line MCF-7 with mTOR inhibitors resulted in an increase in 4E-BP3 protein levels (Fig. 1a). mTOR inhibition also resulted in 4E-BP1 hypophosphorylation, in a concentration-dependent manner (Fig. 1a), as previously reported^17^,^18^. Time-course analysis revealed that 4E-BP3 protein levels increased in a time-dependent manner on mTOR inhibition (Fig. 1b). Next, we examined mRNA levels for each 4E-BP in response to mTOR inhibition (Fig. 1c–f). As observed for the protein, the level of *4E-BP3* mRNA, but not *4E-BP1* or *2*, was increased after treatment with mTOR inhibitors in the three cancer cell lines (Fig. 1c,d and Supplementary Fig. 1a,b). The effect of mTOR inhibitors on *4E-BP3* mRNA level was gradual over the course of 72 h (Fig. 1e,f and Supplementary Fig. 2a). A similar induction of 4E-BP3 was observed in cells subjected to serum or amino acid starvation and in mouse cell lines treated with mTOR inhibitors (Supplementary Fig. 2b,c). Treatment with the transcription inhibitor, actinomycin D, suppressed the increase in *4E-BP3* mRNA in response to mTOR inhibition (Fig. 1g,h), demonstrating that 4E-BP3 induction occurs at the transcriptional level. The induction of *4E-BP3* mRNA occurred in human hepatocellular carcinoma HepG2, but *4E-BP3* mRNA was not detected in three other human cell lines: HEK293T, HeLa and 786-O, even upon mTOR inhibition (Supplementary Fig. 3a). This is explained by the discovery that the *EIF4EBP3* gene is silenced by DNA methylation in those cell lines, and that pretreatment with the DNA-demethylating agent (5-azacitidine) induced *4E-BP3* mRNA on mTOR inhibition (Supplementary Fig. 3b).

Rapamycin is primarily regarded as an mTORC1 inhibitor, and not an mTORC2 inhibitor, because the latter complex is largely resistant to acute rapamycin treatment. Prolonged rapamycin treatment does, however, interfere with the mTORC2 activity by preventing mTORC2 assembly^29^. To show directly that mTORC1, but not mTORC2, induces 4E-BP3, we depleted RAPTOR or RICTOR, the specific components of mTORC1 and mTORC2, respectively^4^,^5^,^6^. RAPTOR depletion, but not that of RICTOR, increased 4E-BP3 expression at both protein and mRNA levels (Fig. 1i,j). Thus, these results demonstrate that 4E-BP3 induction occurs specifically in response to mTORC1 inhibition.

### TFE3 activates *4E-BP3* transcription on mTORC1 inhibition

**Figure 2:**
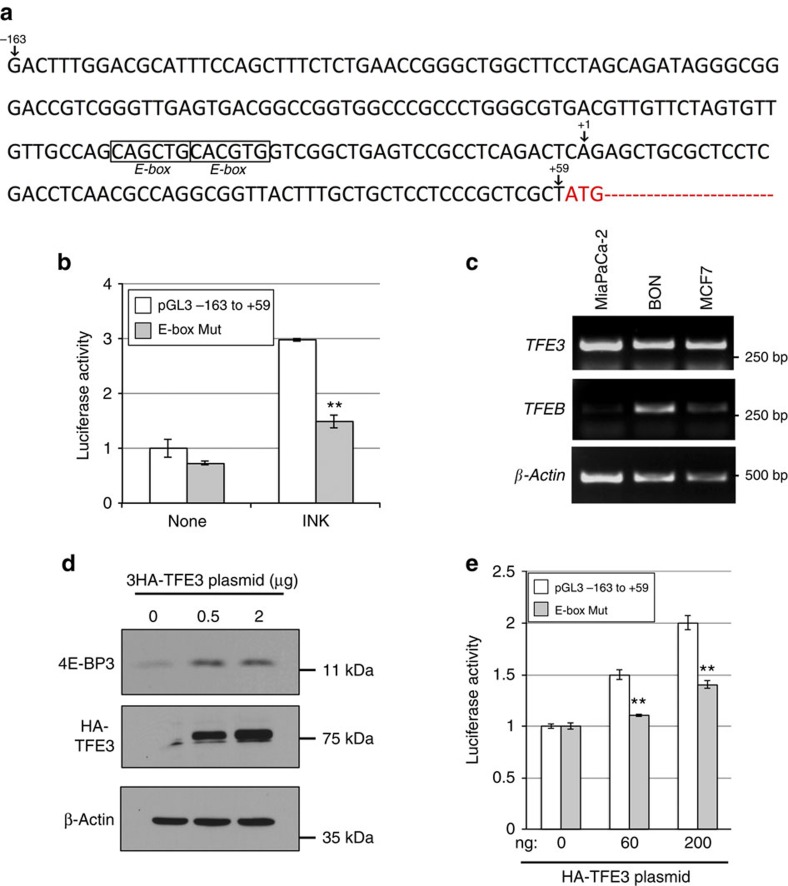
E-boxes function as *cis*-elements for the *EIF4EBP3* promoter activation by TFE3. (**a**) Sequence (−163 to +59) in the *EIF4EBP3* promoter region. Position +1 indicates the information of putative transcription start site (the DBTSS (http://dbtss.hgc.jp)). Protein coding region is shown by red characters. (**b**) E-box mutation reduces *EIF4EBP3* promoter activity. E-box sequences 5′-CAGCTG CACGTG-3′ were changed to 5′-GTCCTG GTGGTG-3′. MiaPaCa-2 cells were transiently transfected with p4E-BP3pro-Luc (−163 to +59) plasmid or its E-boxes mutant and pRL-TK reporter plasmid as a control. After 24 h, cells were treated with 100 nM of INK1341 for 24 h. Relative luciferase activity was measured using Dual-Luciferase Assay kit. Error bars indicate±s.d. (*n*=3). **P<0.01. Statistical significance was determined using two-way analysis of variance (ANOVA). A representative result of three independent experiments is shown. (**c**) *TFE3* and *TFEB* expression in three cell lines. Each mRNA expression was analysed by RT–PCR. (**d**) MiaPaCa-2 cells were transiently transfected with the indicated amount of TFE3 (HA-tag) plasmid. Detection of the proteins was performed by immunoblotting. A representative result of two independent experiments is shown. (**e**) MiaPaCa-2 cells were transiently transfected with the indicated amount of TFE3 (HA-tag) plasmid together with reporter plasmids as shown in **b**. After 48 h, Relative luciferase activity was measured using Dual-Luciferase Assay kit. Error bars indicate±s.d. (*n*=3). **P<0.01. Statistical significance was determined using two-way ANOVA. A representative result of three independent experiments is shown.

To examine how the transcriptional induction of *4E-BP3* is regulated during mTORC1 inhibition, we generated luciferase reporter constructs containing various segments of the *EIF4EBP3* promoter region and the proximal transcribed sequence (Supplementary Fig. 4a, left). Luciferase assay data showed that the −163 to +59 region of the promoter suffices for *4E-BP3* activation following mTORC1 inhibition (Supplementary Fig. 4a, right). *In-silico* analysis of the −163 to +59 sequences revealed that this region harbours two tandem E-boxes (consensus sequence: 5′-CANNTG-3′) that are potential binding sites for TFE3/TFEB^26^,^27^,^30^,^31^,^32^,^33^, in close proximity to the putative transcription start site (Fig. 2a). TFE3 and TFEB are transcription factors (TFs), which are activated upon mTORC1 inhibition^26^,^27^. To determine whether the E-boxes are important for the response to mTORC1 inhibition, we mutated the E-boxes sequences (5′-CAGCTG CACGTG-3′ into 5′-GTCCTG GTGGTG-3′) and examined the activity of the mutated promoter using the luciferase assay. As expected, the mutation of E-boxes reduced by half the activation of the *EIF4EBP3* promoter following mTORC1 inhibition as compared with the wild-type (WT) reporter (Fig. 2b), suggesting that TFE3/TFEB mediates the transcriptional induction of *4E-BP3* by mTORC1.

We examined mRNA expression of *TFE3/TFEB* in the cell lines (MiaPaCa2, BON and MCF-7) in which *4E-BP3* was induced on mTORC1 inhibition (Fig. 1c,g). *TFE3* expression was abundant in the three cell lines, whereas *TFEB* expression was low in two of the cell lines (in MiaPaCa-2 and MCF-7 cells), but present in BON cells (Fig. 2c). Overexpression of TFE3 in MiaPaCa-2 induced the 4E-BP3 protein (Fig. 2d). Accordingly, overexpression of TFE3 increased 4E-BP3 promoter activity in a dose-dependent manner, whereas mutations of the E-boxes impaired this activity (Fig. 2e). Similar results were obtained on overexpression of TFEB, demonstrating that TFEB is also competent to activate *4E-BP3* transcription (Supplementary Fig. 4b,c).

**Figure 3:**
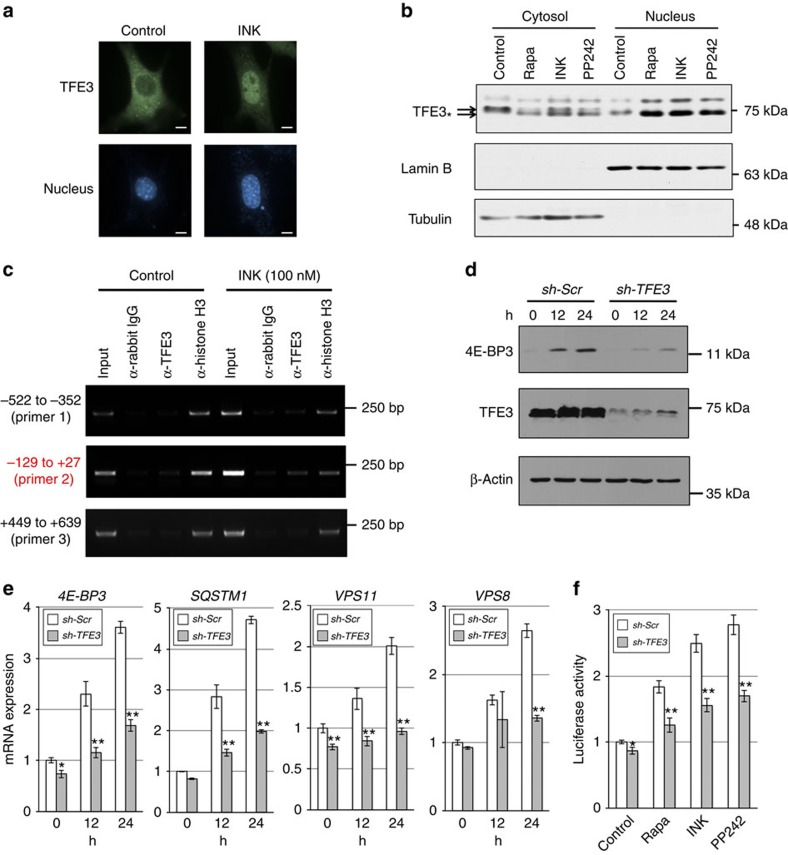
TFE3 mediates the transcriptional induction of *4E-BP3*. (**a**) MiaPaCa-2 cells were treated with 100 nM of INK1341 for 1 h. The cells were fixed and endogenous TFE3 protein in cells was stained with anti-TFE3 antibody and Alexa-Fluor 488. Scale bar, 10 μm. (**b**) MiaPaCa-2 cells were treated with 100 nM of INK1341 for 1 h. Cells were lysed with a Dounce homogenizer. Nuclear and cytoplasmic fractions were separated by centrifugation. Protein levels were determined by immunoblotting. Arrow shows phosphorylated or dephosphorylated form (arrow with star). (**c**) Cells were treated with 100 nM of INK1341 for 1 h. Binding of TFE3 to the E-box region in *EIF4EBP3* promoter was analysed by chromatin immunoprecipitation assay. (**d**,**e**) MiaPaCa-2 cells were transduced with scrambled (sh-Scr) or TFE3 shRNA (sh-TFE3). Protein and mRNA levels were determined by immunoblotting and real-time PCR analysis, respectively. mRNA levels were normalized against β-Actin. Error bars indicate±s.d. (*n*=3). *P<0.05 and **P<0.01. Statistical significance was determined using two-way analysis of variance (ANOVA). (**f**) MiaPaCa-2 cells expressing scrambled or TFE3 shRNA were transiently transfected with reporter plasmids (p4E-BP3pro-Luc (−163 to +59) plasmid and pRL-TK). After 24 h, the cells were treated with rapamycin (100 nM), INK1341 (100 nM) or PP242 (1 μM) for 24 h and then relative luciferase activity was determined as shown in **b**. Error bars indicate±s.d. (*n*=3). *P<0.05 and **P<0.01. Statistical significance was determined using two-way ANOVA. A representative result of three (**a**,**b**) or two independent experiments (**c**–**f**) is shown.

Next, we studied the role of endogenous TFE3 in 4E-BP3 induction using MiaPaCa-2 cells, which express TFE3, but not TFEB (Fig. 2c). As previously reported^26^, immunofluorescence analysis and subcellular fractionation showed that TFE3 accumulated in the nucleus, when cells were treated with mTOR inhibitors or subjected to serum starvation (Fig. 3a,b and Supplementary Fig. 5a–c). Thus, mTORC1 inhibition by pharmacological or nutritional deprivation results in nuclear localization of TFE3.

To determine whether TFE3 directly binds to the *EIF4EBP3* promoter at the E-boxes, we performed a chromatin immunoprecipitation assay. In response to asTORi treatment, TFE3 bound to the DNA segment containing the E-boxes (−129 to +27) of the *EIF4EBP3* promoter, but not upstream (−522 to −352) or downstream (+449 to +639) regions (Fig. 3c), suggesting that TFE3 can directly bind to the E-box region of the *EIF4EBP3* promoter under mTORC1 inhibition. To further confirm that TFE3 is required for the *4E-BP3* induction, we established a stable *TFE3* knockdown cell line. TFE3 knockdown impaired 4E-BP3 induction at the protein level (Fig. 3d) and at the mRNA level as well (Fig. 3e). Similar results were obtained using MCF-7 cells (Supplementary Fig. 5d,e). As expected, TFE3 knockdown also impaired induction of known TFE3/TFEB targets: *SQSTM1*, *VPS11* and *VPS8* (Fig. 3e)^26^,^28^. In contrast, TFE3 knockdown barely affected *GAPDH* or *ATP5G1.* The latter is an mRNA, which is reduced on mTORC1 inhibition (Supplementary Fig. 6)^34^. A luciferase assay demonstrated that TFE3 is required for full activation of the *EIF4EBP3* promoter by mTOR inhibitors (rapamycin, INK1341 or PP242) (Fig. 3f).

To determine whether other TFs may be implicated in the control of 4E-BP3 expression, we screened for candidates that are activated on mTOR inhibition using the TF Activation Profiling Plate Array II FA-1002 (Signosis, CA) and found that, in addition to TFE3, two other TFs (SRY and CBF) are strongly activated (2.5- to ∼3-fold), whereas four other TFs (EGR, Ets, SMAD and RUNX) are activated to a lesser extent (1.5- to ∼2-fold; Supplementary Fig. 7a). We then performed an *in-silico* query for putative binding sites within the *EIF4EBP3* promoter region (−163 to ∼+59) for these 7 TFs using the Jaspar software (http://jaspar.genereg.net) (Supplementary Fig. 7b). The analysis revealed the presence of possible binding sites for RUNX, EGR and Ets. However, the score for these TFs was lower than that for TFE3, suggesting that although other TFs may also participate in the induction of *4E-BP3* during mTORC1 inhibition, TFE3 is likely to be the predominant factor. Taken together, these results demonstrate that TFE3 mediates the transcriptional induction of *4E-BP3* in response to mTORC1 inhibition.

### 4E-BP3 limits cap-dependent translation

**Figure 4:**
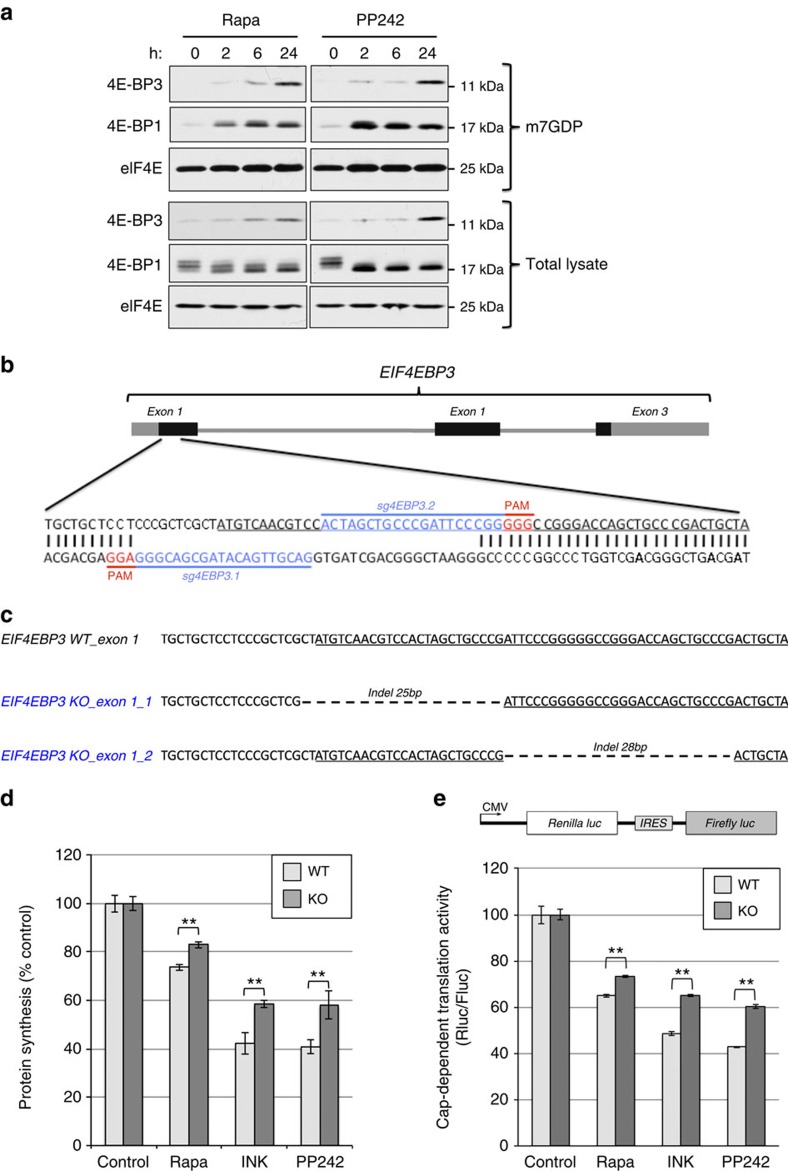
4E-BP3 limits cap-dependent translation during mTORC1 inhibition. (**a**) MiaPaCa-2 cells were treated with mTOR inhibitors (Rapa 100 nM and PP242 1 μM) for the indicated time. Lysates were subjected to m^7^GDP pull-down assay. A representative result of two independent experiments is shown. (**b**) Exon 1 of human *EIF4EBP3* targeted by the Crispr-Cas9 nickase system. Targeted sequences are shown in blue. Protospacer adjacent motif (PAM) is highlighted in red. Protein coding region is underlined. (**c**) Internal deletion induced by two sgRNAs targeting *EIF4EBP3*. Protein coding region is underlined. (**d**) Protein synthesis was measured by a non-radioactive puromycin labelling (SUnSET) assay (see the method). **P<0.01. Statistical significance was determined using two-way analysis of variance (ANOVA). A representative result of three independent experiments is shown. (**e**) A diagram of the bicistronic reporter construct (top). MiaPaCa-2 cells were transfected with the bicistronic reporter plasmid construct for 24 h and then treated with mTOR inhibitors for 12 h. Each luciferase activity was measured using Dual-Luciferase Assay kit (Promega). Error bars indicate±s.d. (*n*=3). **P<0.01. Statistical significance was determined using two-way ANOVA. A representative result of three independent experiments is shown.

To examine the time of the binding of 4E-BP3 to eIF4E during mTOR inhibition, we performed a cap pull-down assay at each time point (see Methods). In this experiment, MiaPaCa-2 cells were treated with rapamycin or PP242 and cell lysate was collected at different time points (2, 6 and 24 h) and subjected to m^7^GDP pull-down assay. Binding of 4E-BP3 to eIF4E was significantly increased after 24 h of mTORC1 inhibition. This is a relatively long time in comparison with 4E-BP1, whose binding to eIF4E increased dramatically as early as 2 h after mTORC1 inhibition (Fig. 4a). Thus, 4E-BP3-mediated control of cap-dependent translation is manifested primarily at later time points of mTORC1 inhibition.

To elucidate the physiological role of 4E-BP3 induction, we generated 4E-BP3 knockout (KO) human MiaPaCa-2 cells using the CRISPR-Cas9 nickase system, which minimizes off-target activity^35^,^36^. Control WT cells were generated using non-targeting guide RNAs (Fig. 4b,c). Sanger sequencing confirmed that two types of frameshift indels were created in the targeted region of *EIF4EBP3* exon1 in the KO cells, but not in the WT cells (Fig. 4b,c). First, we measured protein synthesis on mTOR inhibitor treatments in 4E-BP3 WT and KO cells using a non-radioactive puromycin-labelling assay (SUnSET)^37^. 4E-BP3 KO cells exhibited elevated the protein synthesis as compared with WT cells (Fig. 4d and Supplementary Fig. 8a,b). Next, we investigated the impact of 4E-BP3 on the cap-dependent translation activity using a bicistronic reporter composed of two open reading frames encoding *Renilla* and firefly luciferase separated by the hepatitis C virus (HCV) internal ribosomal entry site (IRES) (Fig. 4e, top)^38^,^39^. Translation of the *Renilla* cistron is cap dependent, whereas translation of the firefly cistron is controlled by the HCV IRES and, therefore, occurs in a cap-independent manner (translation of the firefly cistron is used as an internal control for transfection efficiency)^38^,^39^. On treatment with mTOR inhibitors, cap-dependent translation activity decreased by 50–60% in WT cells, whereas the translation activity was sustained at a higher level (∼10–20% increase) in KO cells (Fig. 4e and Supplementary Fig. 9). The results obtained from the bicistronic reporter assay bolster the findings obtained using the SUnSET assay. In addition, transient expression of WT 4E-BP3 in 4E-BP3 KO cells using a doxycycline (Dox)-inducible construct, fully restored the inhibitory effect of mTOR inhibitors on cap-dependent translation, whereas expression of the 4E-BP3 Y40A mutant that lacks eIF4E-binding activity failed to do so (Supplementary Fig. 10)^24^.

**Figure 5:**
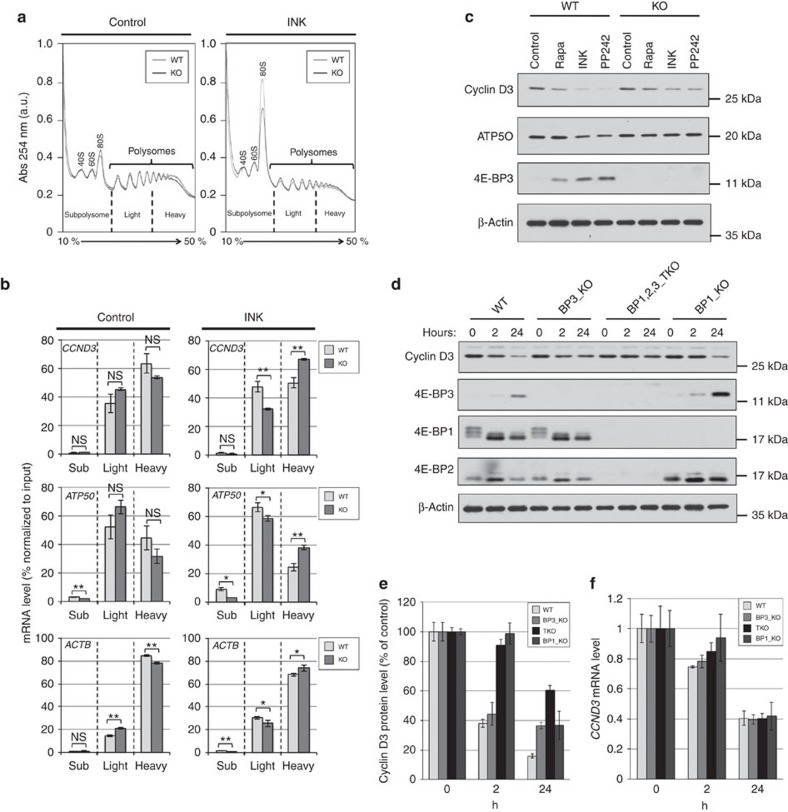
4E-BP3 regulates translation of eIF4E-target mRNAs under prolonged mTORC1 inhibition. (**a**) Polysome profiling in 4E-BP3 WT and KO MiaPaCa-2 cells. The cells were treated with 100 nM INK1341 for 24 h. (**b**) Abundance of each mRNA in heavy, light or subpolysomes was analysed by real-time PCR. Error bars indicate±s.d. (*n*=3). *P<0.05 and **P<0.01. Statistical significance between WT and KO in each fraction (Sub, Light or Heavy) was determined using Student's *t*-test. (**a**,**b**) A representative result of two independent experiments is shown. (**c**) The 4E-BP3 WT or KO MiaPaCa-2 cells were treated with the indicated mTOR inhibitors (Rapa 100 nM, INK1341 100 nM and PP242 1 μM) for 24 h. Each protein expression was determined by immunoblotting. A representative result of three independent experiments is shown. (**d**) 4E-BP3 WT, KO, 4E-BP1,2,3 TKO or 4E-BP1 KO cells were treated with INK1341 (100 nM), for 2 or 24 h. Each protein expression was determined by immunoblotting. A representative result of two independent experiments is shown. (**e**) Cyclin D3 protein expression was quantified by densitometry analysis using ImageJ and normalized by β-Actin level. Error bars indicate±s.d. (*n*=3). (**f**) *CCND3* mRNA level was analysed by real-time PCR. Error bars indicate±s.d. (*n*=3). A representative result of three or two independent experiments is shown.

To further establish the role of 4E-BP3 induction in the control of protein synthesis, we performed polysome profile analysis. Treatment with mTOR inhibitors (most notably active-site mTOR inhibitors) causes a profound translation initiation arrest, readily noted by a dramatic increase in the 80S ribosomal peak^18^,^40^,^41^. The increase in the 80S ribosomal peak and reduction in polysomal load induced by mTOR inhibitors was less pronounced in 4E-BP3 KO cells as compared with their WT counterparts (Fig. 5a). Conversely, ectopic expression of 4E-BP3 in HEK293T, a 4E-BP3-negative cell line (Supplementary Fig. 3), augmented the effect of mTOR inhibitors (Supplementary Fig. 11a,b). Importantly, eIF4E-target mRNAs *cyclin D3* (*CCND3*) and *ATP5O*^17^,^18^,^42^ were retained in heavy polysome fractions in 4E-BP3 KO cells during mTORC1 inhibition as compared with WT (Fig. 5b), indicating that the translation of these mRNAs is preserved in the KO cells. Consistent with these findings, the expression of cyclin D3 and ATP5O proteins was decreased during prolonged mTORC1 inhibition in WT cells, whereas their expression was sustained in 4E-BP3 KO cells (Fig. 5c and Supplementary Fig. 12). Dox-induced expression of 4E-BP3, but not that of the Y40A mutant, in KO cells ameliorated the decrease in cyclin D3 expression resulting from mTORC1 inhibition (Supplementary Fig. 13a,b). To investigate the impact of 4E-BP3, relative to its homologues, on cyclin D3 protein level, we compared 4E-BP3 KO cells with 4E-BP1,2,3 triple KO (TKO) cells or 4E-BP1 KO cells generated by the CRISPR-Cas9 system (Supplementary Figs 14 and 15). We measured cyclin D3 protein levels at early (2 h) and late (24 h) time points of mTOR inhibitor treatments. At 2 h, cyclin D3 protein expression in the 4E-BP3 KO cells was decreased to a level similar to that observed for WT cells, whereas there was a clear difference in cyclin D3 protein expression between 4E-BP3 KO and WT cells at 24 h, at which point the expression of cyclin D3 protein was maintained at much higher levels (twofold) in 4E-BP3 KO cells as compared with WT cells (Fig. 5d,e). By comparison, the levels of cyclin D3 protein in TKO cells at 2 h were unchanged and remained so for 24 h, where a threefold higher cyclin D3 expression was observed in TKO compared with WT cells (Fig. 5d,e). TKO cells also sustained cap-dependent translation activity at much higher level (Supplementary Fig. 16). 4E-BP1 KO cells sustained cyclin D3 protein level at the early time point, but not at the late time point (Fig. 5d,e). Thus, in accordance with its induction level, 4E-BP3 had a significant impact on translation at late time points of mTORC1 inhibition. For *CCND3* mRNA levels, there was no significant difference between the cells, although the mRNA level was reduced in response to treatment with asTORi (Fig. 5f).

### 4E-BP3 is an important effector of mTORC1 in cancer

**Figure 6:**
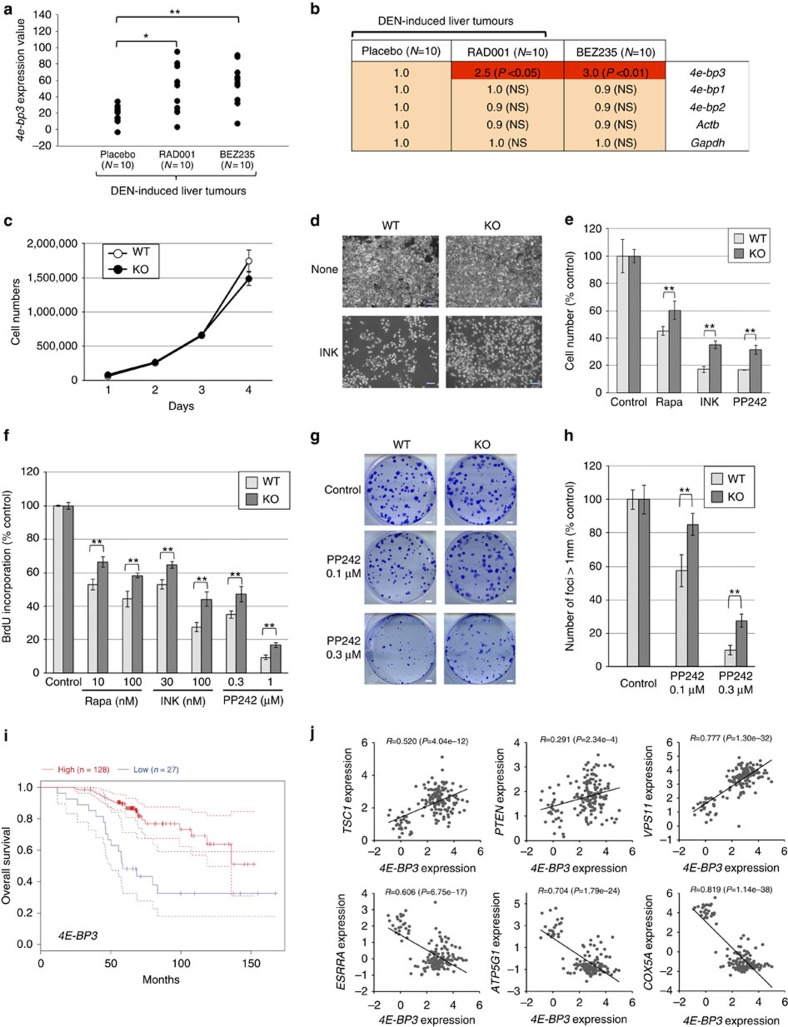
4E-BP3 is an important downstream effector of mTORC1 in cancer. (**a**,**b**) The data set was obtained from the Gene Expression Omnibus (GEO) database www.ncbi.nlm.nih.gov/geo (accession number GSE37129)^43^. Male C57BL/6 mice were injected with diethylnitrosamine (DEN) at 2 weeks. After 44 weeks, mice were treated daily with placebo, RAD001 or BEZ235. (**a**,**b**) Relative change in each gene expression was determined by dividing the average of expression value in drug-treated samples by the average of expression values in placebo (**b**). Statistical significance was determined using one-way analysis of variance (ANOVA). *P<0.05, **P<0.01. (**c**) 4E-BP3 WT or KO MiaPaCa-2 cells were incubated for the indicated days. Live-cell numbers were counted automatically using a BioRad TC10 automated cell counter. (**d**,**e**) 4E-BP3 WT or KO cells were treated with mTOR inhibitors for 72 h. Scale bar, 200 μm. Live cells were counted automatically using a BioRad TC10 automated cell counter. Error bars indicate±s.d. (*n*=3). **P<0.01. (**f**) The WT or KO cells were treated with the mTOR inhibitors for 72 h. Cell proliferation was measured by BrdU incorporation. Error bars indicate±s.d. (*n*=3). **P<0.01. (**g**) 4E-BP3 WT or KO cells were grown on a monolayer and focus formation was determined after 10 days by crystal violet staining. Scale bar, 2 mm. (**h**) Number of foci >1 mm was counted using ImageJ. Results represent the mean cell number relative to control (set to 100%)±s.d. (*n*=3). **P<0.01. Statistical significance was determined using two-way ANOVA. (**c**–**h**) A representative result of three independent experiments is shown. (**i**) The data set was obtained from the Gene Expression Omnibus (GEO) database, www.ncbi.nlm.nih.gov/geo (accession number GSE9893)^45^. Kaplan–Meier analyses of overall survival of 155 breast cancer patients stratified by high (red) and low (blue) *4E-BP3* (Log rank test *P*-value <0.0001). Dotted lines indicate 95% confidence intervals. (**j**) Correlation between the expression of *4E-BP3* and the following genes: *TSC1*, *PTEN*, *VPS11*, *ESRRA*, *ATP5G1* and *COX5A*, in human breast cancer tissues (accession number GSE9893). The correlation coefficient was determined using Spearman's coefficient analysis.

Next, we wished to investigate the possible role of 4E-BP3 downstream of mTORC1 in cancer. To this end, we analysed an existing microarray data set deposited by Thomas *et al.*^43^. This data set documents gene expression profiles in mouse liver tumours (*in vivo*) treated with mTOR-targeting drugs, using Illumina's gene chip (mouse ref8 v2), which contains specific probes for mouse *4e-bp3* without detecting the *Ankhd1-Eif4ebp3* fusion transcript^44^. The data set is therefore appropriate for studying mouse *4e-bp3* expression. Thomas *et al.*^43^ examined gene expression patterns in the chemical carcinogen diethylnitrosamine-induced liver tumours in mice administrated with the allosteric mTORC1 inhibitor RAD001 (rapalog) and/or active-site mTOR/phosphoinositol 3-kinase dual inhibitor BEZ235. Consistent with the results obtained with cultured cells (Fig. 1), *4e-bp3* mRNA expression, but not *4e-bp1* and *2*, was significantly induced in tumours administered with each drug (RAD001 (2.5-fold) or BEZ235 (3.0-fold) alone) (Fig. 6a,b). The combination of RAD001 and BEZ235 on *4e-bp3* expression had no additive effect (Supplementary Fig. 17a,b). Thus, a single treatment of mTORC1-targeting drug was sufficient to induce *4e-bp3* expression *in vivo*.

Next, we investigated the ability of 4E-BP3 to mediate the anti-proliferative effects of mTOR inhibitors, by comparing human WT cells with 4E-BP3 KO cells. There was no significant difference in proliferation between WT and 4E-BP3 KO cells under normal growth conditions (Fig. 6c). In contrast, on mTOR inhibition (rapamycin, INK1341 and PP242), 4E-BP3 KO cells exhibited faster proliferation than WT cells (Fig. 6d–f). In addition, Dox-induced overexpression of 4E-BP3, but not the Y40A mutant, re-sensitized 4E-BP3 KO cells to mTOR inhibitors (Supplementary Fig. 18a,b), demonstrating that the drug resistance of the 4E-BP3 KO cells can be attributed to 4E-BP3-mediated cap-dependent translation. 4E-BP3 KO cells also exhibited increased clonogenic growth in the presence of PP242 as compared with WT cells (Fig. 6g,h). Thus, 4E-BP3 functions as a negative regulator of cell proliferation under prolonged mTORC1 inhibition, demonstrating that 4E-BP3 is an important downstream effector of mTORC1 in cancer cells.

Finally, we analysed the relationship between 4E-BP3 expression and poor cancer prognosis using the data set of human breast cancer patients deposited by Chanrion *et al.*^45^. Similar to the Thomas *et al.*^43^ study, this data set was appropriate for studying *4E-BP3* expression, as it contained specific probes for *4E-BP3*. High *4E-BP3* levels, which was determined by the distribution of the *4E-BP3* expression values (Supplementary Fig. 19), associated with longer overall survival of the patients as compared with that of low *4E-BP3* levels (Fig. 6i). We analysed the expression of *4E-BP3* and several genes relevant to the mTORC1 pathway based on the data deposited by Chanrion *et al.*^45^. Importantly, *4E-BP3* expression positively correlated with *TSC1* and *PTEN* levels, both of which are negative regulators of mTORC1, and *VPS11*, which is also upregulated following mTORC1 inhibition (Figs 3e and 6j). In contrast, *4E-BP3* levels negatively correlated with the expression of *ESRRA*, *ATP5G1* and *COX5A*, which are induced by mTOR activation via the peroxisome proliferator-activated receptor-γ co-activator-α and yin-yang 1 transcriptional complex (Fig. 6j)^34^. Thus, *4E-BP3* represents a potential prognostic marker that reflects the activation status of mTORC1 pathway in cancer. Surprisingly, the frequency of distant metastasis and lymph node metastasis (pN1) was significantly higher in the *4E-BP3* low group (Supplementary Fig. 20), suggesting 4E-BP3 expression level may predict metastatic potential of tumours in addition to mTORC1 activation status.

## Discussion

This study describes a novel role for 4E-BP3 in the control of protein synthesis in the context of prolonged inhibition of mTORC1. We show that 4E-BP3, but not 4E-BP1 or 4E-BP2, is transcriptionally induced during prolonged mTORC1 inhibition (Fig. 1). The mTORC1-regulated transcription factor TFE3 mediates the *4E-BP3* induction effect (Figs 2 and 3). 4E-BP3 suppresses translation of eIF4E-target mRNAs under prolonged mTORC1 inhibition (Figs 4 and 5). In addition, 4E-BP3 is induced in tumours treated with mTOR inhibitors and evokes anti-proliferative effects (Fig. 6). These results demonstrate that 4E-BP3 is an important downstream effector of mTORC1 under prolonged mTORC1 inhibition and, thereby, may serve as a predictor of anti-mTOR drug efficacy in cancer therapy.

mTORC1 controls cap-dependent translation via phosphorylation of 4E-BP1 and 2 (refs 1, 8, 10). 4E-BP1 and 4E-BP2 are rapidly phosphorylated by mTORC1 on nutrient stimulation^9^,^46^ and they are swiftly dephosphorylated on mTORC1 inhibition (Figs 1a,b and 4a). In contrast, 4E-BP3 is weakly phosphorylated by mTORC1 (ref. 25), but its amount becomes elevated slowly after mTORC1 inhibition (Figs 1a–f and 4a). Accordingly, 4E-BP3 represses translation of eIF4E-target mRNAs after prolonged inhibition of mTORC1 (Fig. 5b–d). Thus, 4E-BP3 induction appears to be important in maintaining translational repression under prolonged mTORC1 inhibition, perhaps acting as a safeguard mechanism to prevent the reactivation of translation in conditions where nutrient availability remains low. Our data also show that 4E-BP2 expression decreases at later times following mTOR inhibitor treatments (Figs 1a,b and 4d, and Supplementary Fig. 21) at a time when 4E-BP3 increases. Interestingly, cells with acquired resistance to mTOR inhibitors demonstrated reduction in 4E-BP1 and 2 protein expression^17^. It is possible that 4E-BP3 is elevated to counterbalance this effect to maintain translation repression under prolonged mTORC1 inhibition.

We show that the transcriptional induction of *4E-BP3* on mTORC1 inhibition is critical to elevate 4E-BP3 protein levels (Figs 1). Interestingly, the increase in protein levels of 4E-BP3 appears greater than the increase in mRNA levels (Supplementary Fig. 23). Given that the induction in 4E-BP3 protein levels during mTORC1 inhibition inversely correlates with a decrease in 4E-BP2 expression, or is more apparent in cells depleted of 4E-BP1 (Figs 1a,b and 5d), this suggests that the *4E-BP3* mRNA is also under translational control, or that 4E-BP3 is stabilized by its increased association with eIF4E^47^. Assessing 4E-BP3 protein levels in cancers with different 4E-BP1 amounts could further address a recent study demonstrating that lower 4E-BP1 expression is associated with hightened sensitivity to the phosphoinositol 3-kinase/mTOR-targeting drugs in prostate cancer^48^. Although we find that 4E-BP3 is critical in mediating cell proliferation inhibition during prolonged mTORC1 inhibition, 4E-BP3 was not detected in cell lines such as HEK293T, 786-O and HeLa. However, pretreatment with the DNA demethylating agent 5-azacitidine induced 4E-BP3 mRNA on treatment with mTOR inhibitors (Supplementary Fig. 3a,b), suggesting that the *EIF4EBP3* gene is silenced by DNA methylation in certain cell lines. It is possible that DNA methylation of the *EIF4EBP3* gene also occurs in different cancers. Furthermore, 4E-BP3 may be regulated by not only induction but also by phosphorylation^24^,^25^. Overexpression of 4E-BP3 has a weak effect on polysome formation, whereas it augments polysome reduction in cells exposed to mTOR inhibitors (Supplementary Fig. 11). Thus, the function of 4E-BP3 can be under several regulatory mechanisms that merit further study.

eIF4E is amplified in cancer cell lines, which are selected for resistance to mTOR inhibitors^49^,^50^. In addition, it has also been reported that the eIF4E/4E-BP ratio is an important factor that determines sensitivity of cancer cells to asTORi^17^. We show here that 4E-BP3 is induced through TFE3 during mTORC1 inhibition, and that it contributes to the anti-proliferative effects of mTOR inhibitors (Figs 2, 3 and 6a–h). Importantly, *4E-BP3* may be upregulated in several cancer types (for example, RCC or PNET) for which the mTORC1 inhibitors (the rapalogs, temsirolimus and everolimus) have been approved for treatment^12^,^13^,^14^. For example, data obtained from the cBioPortal (http://www.cbioportal.org)^51^,^52^ show that the *EIF4EBP3* locus is amplified in 17% of clear cell RCC (Supplementary Fig. 22a). In addition, TFE3 is hyperactivated in ∼30–50% of paediatric RCC, which is referred to as Xp11.2 translocation RCC, and in alveolar soft part tissue sarcoma, due to chromosomal rearrangement generating several gene fusions including PRCC-TFE3 and ASPL-TFE3 (refs 53, 54, 55, 56, 57, 58, 59). We find that both PRCC-TFE3 and ASPL-TFE3 positively correlate with increased activation of the *EIF4EBP3* promoter relative to TFE3 (Supplementary Fig. 22b,c), suggesting that *4E-BP3* is upregulated in Xp11.2 RCCs. Given that 4E-BP3 elevation sensitizes cells to mTORC1 inhibition (Fig. 4d–h and Supplementary Fig. 18), it is conceivable that 4E-BP3 is a positive effector of mTOR-targeted drugs for the treatment of these types of RCC. Elevation of *4E-BP3* has been also observed in multiple endocrine neoplasia type 1 (MEN1)-mutated patients^60^, which frequently develop PNETs^61^. It is noteworthy that MEN1-mutated PNET is associated with prolonged survival relative to PNET patients without the MEN1 mutations. Similarly, breast cancer patients with high *4E-BP3* expression have better prognosis^61^ (Fig. 6i). Thus, 4E-BP3 elevation may be a predictor of clinical outcome.

4E-BP1 has been extensively studied and its expression and phosphorylation levels have been used as surrogate markers of tumour malignancy in preclinical^17^,^19^,^43^ and clinical settings^20^,^23^,^62^,^63^. Our study offers the novel idea of using 4E-BP3 as a surrogate marker for tumour development for certain cancers. More importantly, it has been very difficult to use phospho-specific antibodies for immunostaining of fresh and paraffin-embedded tumour specimens, because of the lability of the phospho-group and poor selectivity^64^. Thus, the possibility of using an antibody against total 4E-BP3 may provide a clear advantage in monitoring the efficacy of mTOR-targeted drug therapies.

## Methods

### Reagents

We used 1:1,000 dilution for all antibodies. Two antibodies (home-made antibody number 1842 and HPA045537 (Sigma, St Louis, MO) against human 4E-BP3 were used. Antibody against human TFE3 (A302-622A) was purchased from Bethyl (Montgomery, TX). Antibodies against 4E-BP1(9644), 4E-BP2(2845), rpS6, phospho-rpS6 (Ser240/244) (2215), Lamin B1 (13435) and α-Tubulin (2144S) were purchased from Cell Signaling Technology (Danvers, MA). Antibodies against eIF4E (610270) and β-Actin (A5441) were from Sigma. Raptor antibody was from Millipore (Billerica, MA). Rictor antibody was from Bethyl. Horseradish peroxidase-conjugated anti-rabbit IgG and anti-mouse IgG were from Amersham Biosciences (Baie d'Urfé, QC, Canada). PP242 and INK1341 were provided by Intellikine (La Jolla, CA, USA).

### Cell cultures

Human pancreatic cancer cell lines MiaPaCa-2, PANC-1, breast cancer MCF-7, embryonic kidney cell HEK293T, cervical cancer HeLa, hepatocellular carcinoma HepG2 and mouse fibroblast NIH3T3 cell lines were obtained from ATCC and cultured in DMEM medium supplemented with 10% FBS (Invitrogen), 2 mM L-glutamine and 100 units per ml penicillin/streptomycin (all from Invitrogen) at 37 °C and 5% CO_2_. Mouse embryo fibroblasts (p53−/−) were cultured as described^18^. Human PNET cell line BON^65^,^66^ was a gift from Dr Pyronnet (France) and cultured in DMEM/F12 50:50 medium (Invitrogen).

### Virus infections

Lentiviral vectors were from Sigma. Short hairpin RNA (shRNA) vector accession numbers are: human TFE3 (Sigma: TRCN0000019980) and the Non-Target shRNA Control (Sigma: SHC002). Raptor and rictor shRNAs were described^18^. shRNA vectors were co-transfected into HEK293T cells with the lentivirus packaging plasmids PLP1, PLP2 and PLP-VSVG (Invitrogen) using Lipofectamine 2000 (Invitrogen). Supernatant was collected at 48 and 72 h post transfection, passed through a 0.45-μm nitrocellulose filter and applied on target cells with polybrene (5 μg ml^−1^). Cells were re-infected the next day and selected with puromycin for 48 h (1 μg ml^−1^, Sigma).

### RT–PCR and real-time PCR analysis

RNA was isolated from the indicated cell lines using Trizol (Invitrogen) according to the manufacturer's instructions. RT–PCR and real-time PCR reactions were carried out using SuperScript III First-Strand Synthesis System (Invitrogen) and iQ SYBR Green Supermix (Bio-Rad, Hercules, CA) according to the manufacturer's instructions. Primers are listed in Supplementary Fig. 24.

### Western blot analysis

Briefly, cells were lysed in 1 × SDS sample buffer and protein concentrations of the lysates were measured with a Bio-Rad protein assay kit. Equal amounts of proteins were resolved on 8 or 15% polyacrylamide-SDS gels and transferred by electroblotting to a nitrocellulose membrane. Membranes were probed with antibodies, as indicated, and processed with an enhanced chemiluminescence detection system (GE Healthcare Bio-Sciences Corp., Tokyo, Japan). Uncropped blots are shown in Supplementary Fig. 25.

### Subcellular fractionation

MiaPaCa-2 cells were harvested, disrupted in buffer A (50 mM Tris-HCl pH 7.5, 5 mM EDTA and 1 mM dithiothreitol (DTT)) using a Dounce-type homogenizer and then centrifuged at 1,000 *g* for 10 min to obtain a nuclear pellet. The resulting supernatant was mixed with an equal volume of 2 × SDS buffer to prepare the cytosolic fraction. The nuclear pellet was washed using the buffer A and centrifuged at 1,000 *g* again. The final pellet was suspended in 50 μl of the buffer A and lysed with an equal volume of 2 × SDS buffer.

### Reporter assays

Cells were transfected with a firefly luciferase-containing reporter plasmid (p4E-BP3 promoter) and *Renilla* luciferase-containing plasmid pRL-TK (Promega) as an internal control. Relative activity of firefly luciferase to *Renilla* luciferase (mean±s.d. of triplicate determinations) was determined using the Dual-Glo Luciferase Assay System and GLOMAX 20/20 (Promega). For the bicistronic reporter assay, the *Renilla*–HCV IRES–firefly luciferase reporter plasmid (pCMV/Rluc/HCVIRES/Fluc) was used^67^. For the luciferase reporter assays, MiaPaCa-2 cells were seeded in 24-well plates and transfected with 0.1 μg per well of pCMV/Rluc/HCVIRES/Fluc using Lipofectamine 2000 (Invitrogen). Ratios of Rluc/Fluc activity were calculated as a measure of cap-dependent translation.

### Immunofluorescence staining

Cells on a polylysine-coated cover slip were fixed and permeabilized for 10 min in PBS with 4% paraformaldehyde and 0.1% Triton X-100. After blocking for 1 h in PBS with 10% BSA, cells were incubated with anti-TFE3 antibody and subsequent secondary antibody (Alexa-Fluor 488-conjugated anti-rabbit IgG (1:1,000) (Invitrogen) in PBS with 1.5% BSA for 1 h at room temperature.

### Chromatin immunoprecipitation assay

The assay was performed using the SimpleChIP Enzymatic Chromatin IP kit (Cell Signaling Technology) and antibody to TFE3. The promoter region of human *EIF4EBP3* was amplified from immunoprecipitated genomic DNA with the following primers: primers for amplification of −522 to −352 region: forward 5′-CTTAGCCTCCCAAAGTGCTG-3′ and reverse 5′-GCCAAAGTCACACATCTTGC-3′; primers for amplification of −129 to +27 region: forward 5′-GGCTGGCTTCCTAGCAGATA-3′ and reverse 5′-GGCGTTGAGGTCGAGGAG-3′; primers for amplification of +449 to +639 region: forward 5′-GGCTGGCTTCCTAGCAGATA-3′ and reverse 5′-GGCGTTGAGGTCGAGGAG-3′.

### Cap (m^7^GDP) pull-down assay

Cap pull-down assay was carried out as described^18^. Briefly, cells were lysed in the cap pull-down buffer (50 mM HEPES-KOH pH 7.5, 150 mM KCl, 1 mM EDTA, 2 mM DTT and 1% Triton X-100) containing protease and phosphatase inhibitors. Protein extract (1 mg) was incubated for 2 h at 4 °C with m^7^GDP-agarose beads^18^. Beads were washed five times with the cap pull-down buffer and eluted by boiling in 1 × SDS sample buffer for 5 min. m^7^GDP-bound proteins were visualized by western blotting.

### Polysome analysis

Polysome profile analysis was carried out as previously described^18^. Briefly, cells were cultured in 15 cm dishes and treated with INK1341 (100 nM) for 24 h. Cells were washed with cold PBS containing 100 μg ml^−1^ cycloheximide, collected and lysed in a hypotonic lysis buffer (5 mM Tris-HCl pH 7.5, 2.5 mM MgCl_2_, 150 mM KCl, 100 μg ml^−1^ cycloheximide, 2 mM DTT and 1% Triton X-100). Lysates were loaded onto 10–50% sucrose density gradients (20 mM HEPES-KOH pH 7.6, 100 mM KCl and 5 mM MgCl_2_) and centrifuged at 35,000 r.p.m. for 2 h at 4 °C. Gradients were fractionated and optical density at 254 nm was continuously recorded using an ISCO fractionator (Teledyne ISCO, Lincoln, NE).

### Generating human *EIF4EBP3* KO cells using Crispr-Cas9 nickase system

Cas9 D10A coding plasmid (Addgene: pX461) was digested with BbsI and ligated with annealed oligonucleotides corresponding to small guide RNAs (sgRNAs) targeting specific genomic loci. An extra G was added for sgRNAs lacking a 5′G for U6 transcriptional initiation. MiaPaCa-2 cells were grown in DMEM with 10% FBS and a penicillin/streptomycin mix (100 units ml^−1^ and 100 μg ml^−1^, respectively). MiaPaCa-2 cells (3 × 10^5^) were transfected with sgRNA-Cas9-encoding plasmids (0.5 μg of sgRNA1-Cas9 and 0.5 μg of sgRNA2-Cas9) using Lipofectamine 2000 according to the manufacturer's protocol (Invitrogen). For 4E-BP3 WT cells, MiaPaCa-2 cells were transfected with non-sgRNA-Cas9 nickase-encoding plasmid. After 24 h, the cells were collected and seeded at 1 cell per well into 96-well plate. Disruption of the *EIF4EBP3* exon 1 gene in the KO candidate cells was determined by Sanger sequencing.

### Cell proliferation assay

For BrdU incorporation assay (Cell Proliferation ELISA BrdU kit, Roche), cells were seeded in 96-well plates (500 cells per well) and maintained as indicated in the figure legends. Absorbance at 450 nm (reference wavelength 690 nm) was measured using a Varioskan microplate reader (Thermo Electron Corporation, Waltham, MA). For Trypan Blue exclusion, cells were seeded in 6-well plates (100,000 cells per well) overnight and maintained under conditions indicated in the figure legends. Live-cell numbers were automatically counted by a BioRad TC10 automated cell counter.

### Colony formation assay

Cells were plated in 6-well plates at 200 cells per well and the next day the cells were incubated in the absence or presence of PP242. Fresh media containing PP242 was added every 3 days. After 10 days, cells were fixed in methanol and stained with 0.5% crystal violet.

### Analyses of microarray array data on mouse and human tumour specimens

Details of the experimental conditions and normalization of expression value of each gene were published^43^,^45^. The normalized expression values deposited in the Gene Expression Omnibus (GSE37129 and GSE9893) were used for gene expression analyses.

### SUnSET assay

The SUnSET assay was used to monitor the rate of protein synthesis^37^. Briefly, 20 min before harvesting the cells, puromycin was added to culture medium at 2 mg ml^−1^. As a control, cycloheximide was added at 100 μg ml^−1^ at 5 min before puromycin addition, resulting in complete blockade of protein synthesis. Cell extracts were then processed for western blotting using antipuromycin antibody.

### Statistical analysis

Error bars for all data represent s.d. from the mean. *P*-values were calculated using two-tailed Student's *t*-test, one-way or two-way analysis of variance as shown in each legend.

### Data availability

The data that support the findings of this study are available from the authors on request.

## Additional information

**How to cite this article**: Tsukumo, Y. *et al.* Translation control during prolonged mTORC1 inhibition mediated by 4E-BP3. *Nat. Commun.* 7:11776 doi: 10.1038/ncomms11776 (2016).

## Supplementary Material

Supplementary Information Supplementary Figures 1 - 25
