# Supplementary material for: Translation control during prolonged mTORC1 inhibition mediated by 4E-BP3
Source: Nat Commun. 2016 Jun 20;7:11776. doi: 10.1038/ncomms11776 (PMC4915159; doi:10.1038/ncomms11776)
Supplement: Supplementary Information — Supplementary Figures 1 - 25 [file ncomms11776-s1.pdf]

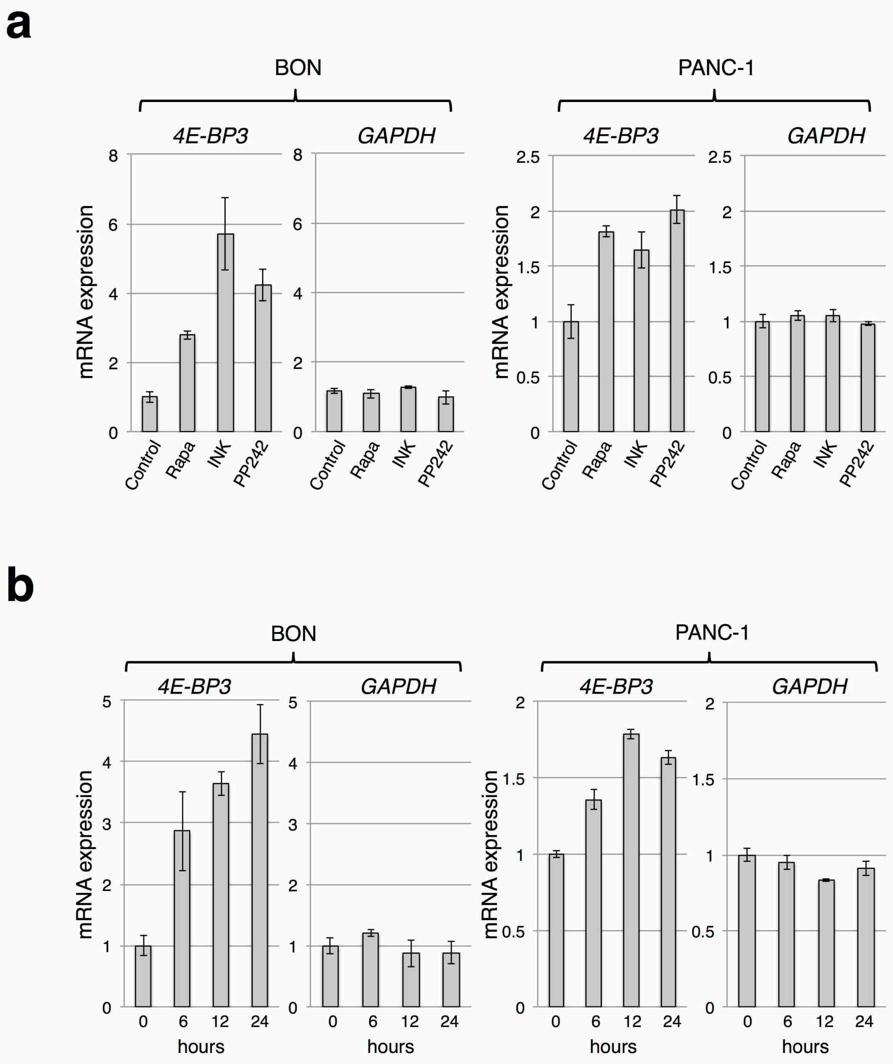

Supplementary Figure 1. qPCR analysis of 4E-BP3 expression in BON and PANC-1 cells

(**a**) BON or PANC-1 cells were treated with rapamycin (100 nM), INK1341 (100 nM), PP242 (1 μM) for 24 hr. Expression of *4E-BP3* was analyzed by qRT-PCR. Expression of *4E-BP3* or *GAPDH* was normalized to *β-actin*. Error bars indicate ± SD (n=3).

(**b**) BON or PANC-1 cells were treated with INK1341 (100 nM) for the indicated times. Expression of *4E-BP3 or GAPDH* expression was analyzed as described in (a).

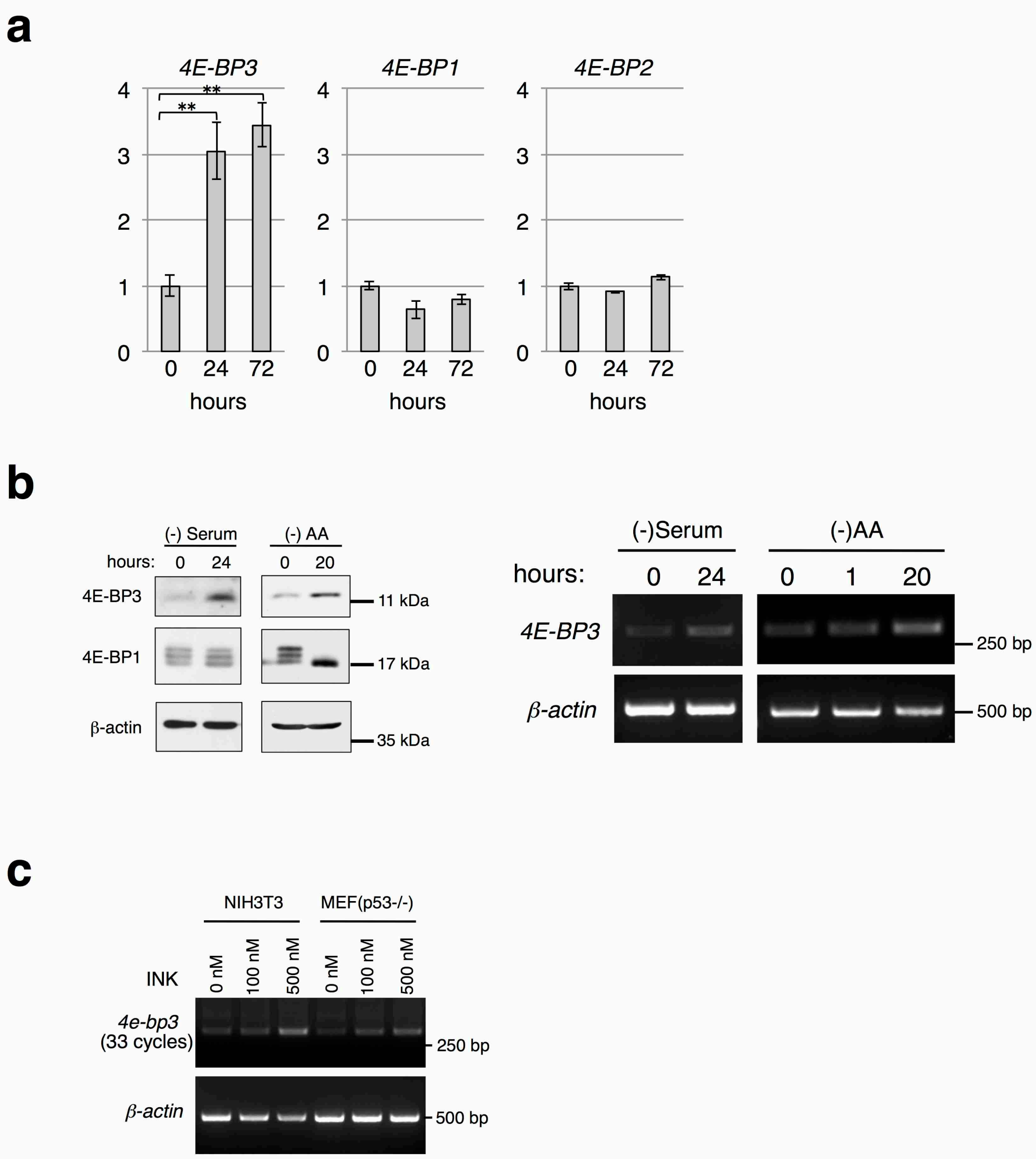

Supplementary Figure 2. 4E-BP3 is also induced by nutrient starvation

(**a**) MiaPaCa-2 cells were treated with INK (100 nM) for 24 hr or 72 hr. Expression of each *4E-BP* in MiaPaCa-2 cells was analyzed by real time PCR. Relative level of 4E-BPs was measured by normalizing with *β-actin*. Error bars indicate ± SD (n=3). **p<0.01. The statistical difference between each group was determined using one-way ANOVA.

(**b**) MiaPaCa-2 cells were incubated without serum or amino acids for the indicated times. Expression of the indicated proteins or mRNAs was determined by immunoblotting or RT-PCR.

(**c**) NIH3T3 or immortalized p53 null MEF (p53-/-) cells were treated with INK1341 for 24 hr. *4e-bp3* expression was analyzed by RT-PCR.

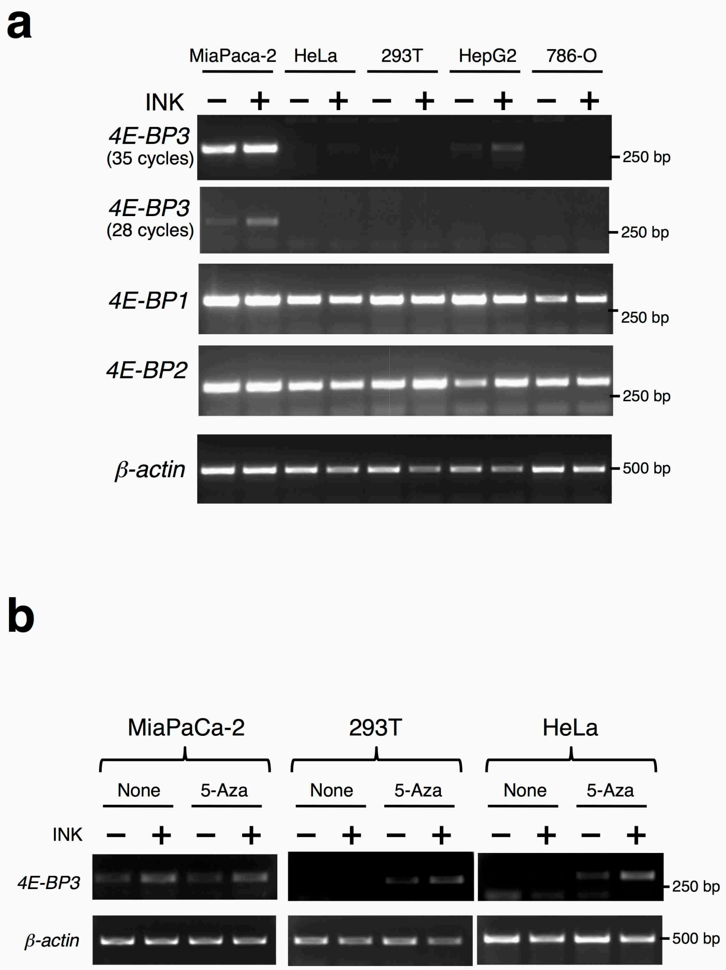

Supplementary Figure 3. 4E-BP3 is not detected in HeLa, 293T and 786-O cells

(**a**) The indicated cell lines were treated with INK (100 nM) for 24 hr. Expression of each *4E-BP* was analyzed by RT-PCR.

(**b**) The indicated cell lines were pretreated with 5-azacitidine (5 μM) for 48 hr and then treated with INK (100 nM) for 24 hr. Expression of the indicated mRNAs were determined by RT-PCR.

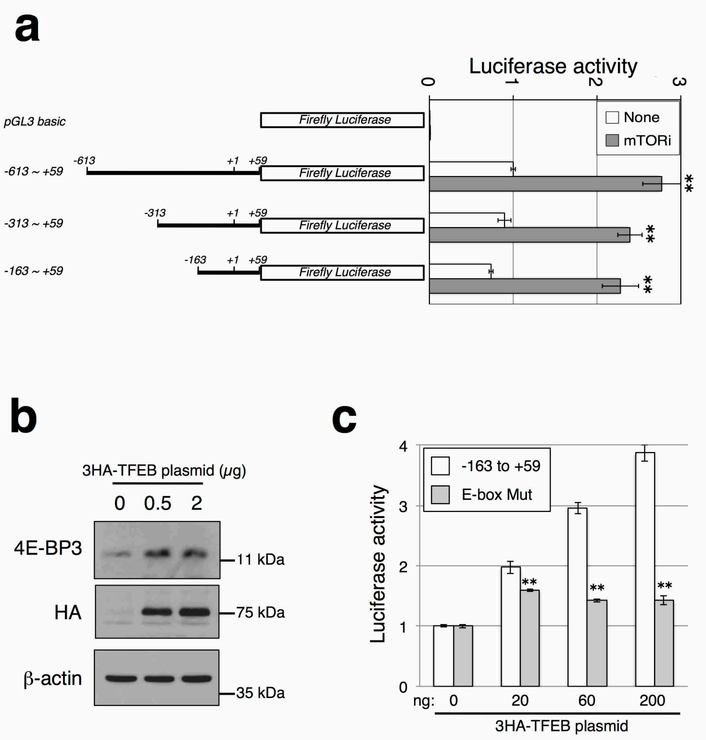

Supplementary Figure 4. TFEB overexpression also activates 4E-BP3 transcription

(**a**) Constructs and relative luciferase activities of human *EIF4EBP3* promoter reporter genes. Position +1 indicates the information of putative transcription start site (TSS) (the DBTSS (http://dbtss.hgc.jp)). MiaPaCa-2 cells were transiently transfected with p4E-BP3pro-Luc plasmid and pRL-TK reporter plasmid as a control. After 24 hr, cells were treated with 100 nM of INK1341 for 24 hr. Relative luciferase activity was measured using dual luciferase assay kit. Error bars indicate ± SD (n=3). **p<0.01. The statistical difference between each group was determined using Student t-test.
(**b**) MiaPaCa-2 cells were transiently transfected with the indicated amounts of TFEB (HA-tag) plasmid.
(**c**) MiaPaCa-2 cells were transiently transfected with the indicated amount of TFEB (HA-tag) plasmid together with reporter plasmids (4E-BP3pro and pRL-TK). After 48 hr, relative luciferase activity was measured using dual luciferase assay kit. Error bars indicate ± SD (n=3). **p<0.01. The statistical difference between each group was determined using two-way ANOVA.

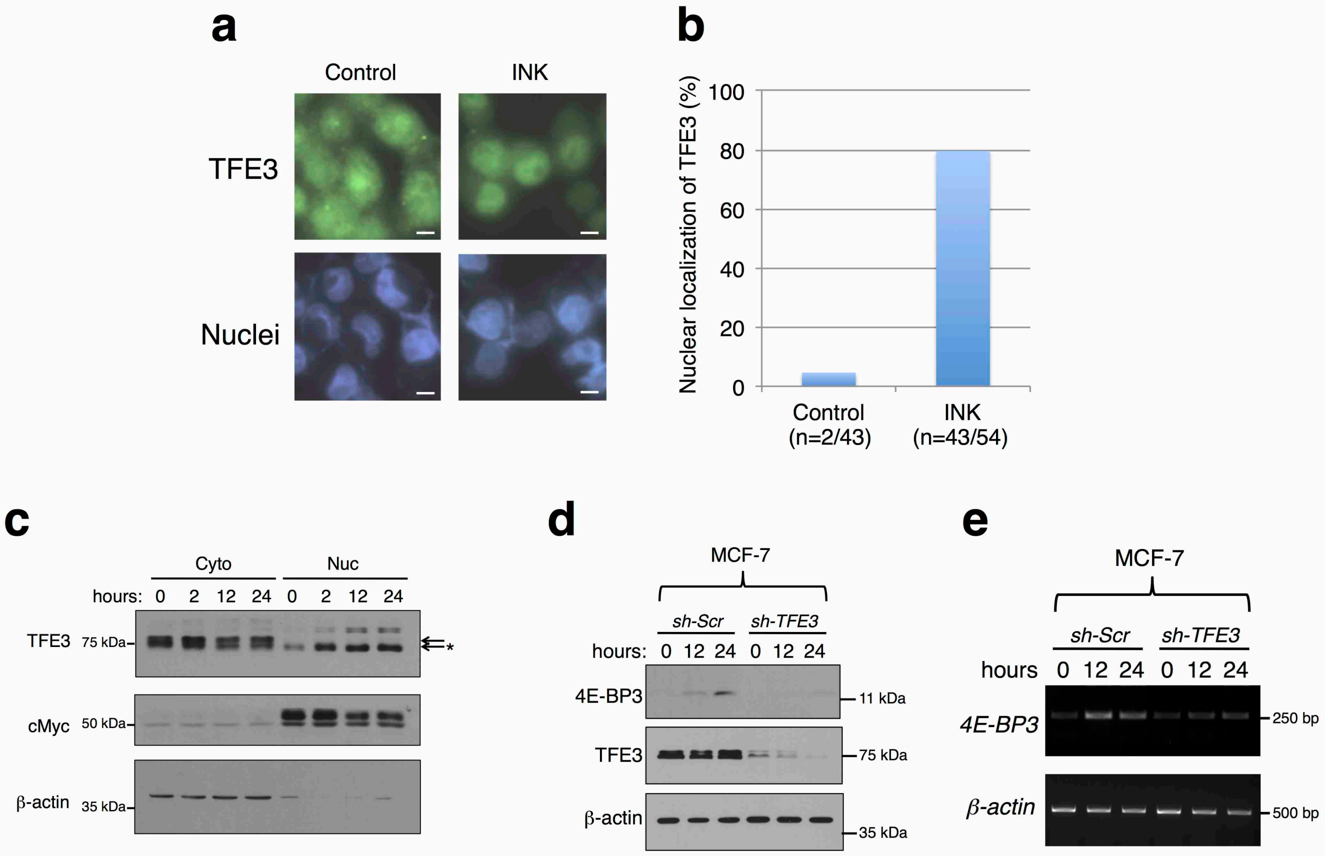

Supplementary Figure 5. Nuclear translocation of TFE3 upon mTOR inhibitor treatment

(**a**) MiaPaCa-2 cells were treated with 100 nM of INK1341 for 1 hr. The cells were fixed and endogenous TFE3 protein in cells was stained with anti-TFE3 antibody and Alexa-fluor 488. scale bar=10 μm.

(**b**) The ratio of cell numbers with nuclear staining of TFE3. Control (n= 43), INK treatment (n=54).

(**c**) MiaPaCa-2 cells were incubated under serum-starved conditions for the indicated times. Cells were destructed with downce homogenizer and then nuclear or cytoplasmic fraction was separated by centrifugation. Each protein level was determined by immunoblotting.

(**d,e**) MCF-7 cells were transduced with scrambled (control sh-Scr), or TFE3 shRNA (sh-TFE3). Each protein or mRNA was determined by immunoblotting or RT-PCR analysis.

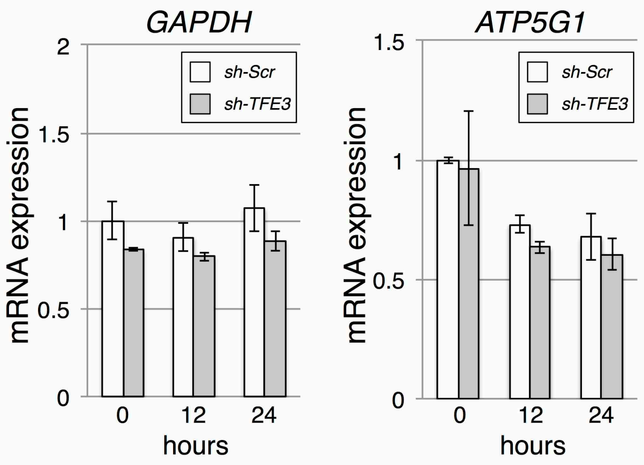

Supplementary Figure 6. GAPDH and ATP5G1 expression in TFE3 knockdown cells

(**a**) MiaPaCa-2 cells were transduced with scrambled (sh-Scr), or TFE3 shRNA (sh-TFE3). mRNA levels were determined by real-time PCR analysis and normalized against *β-actin*. Error bars indicate ± SD (n=3).

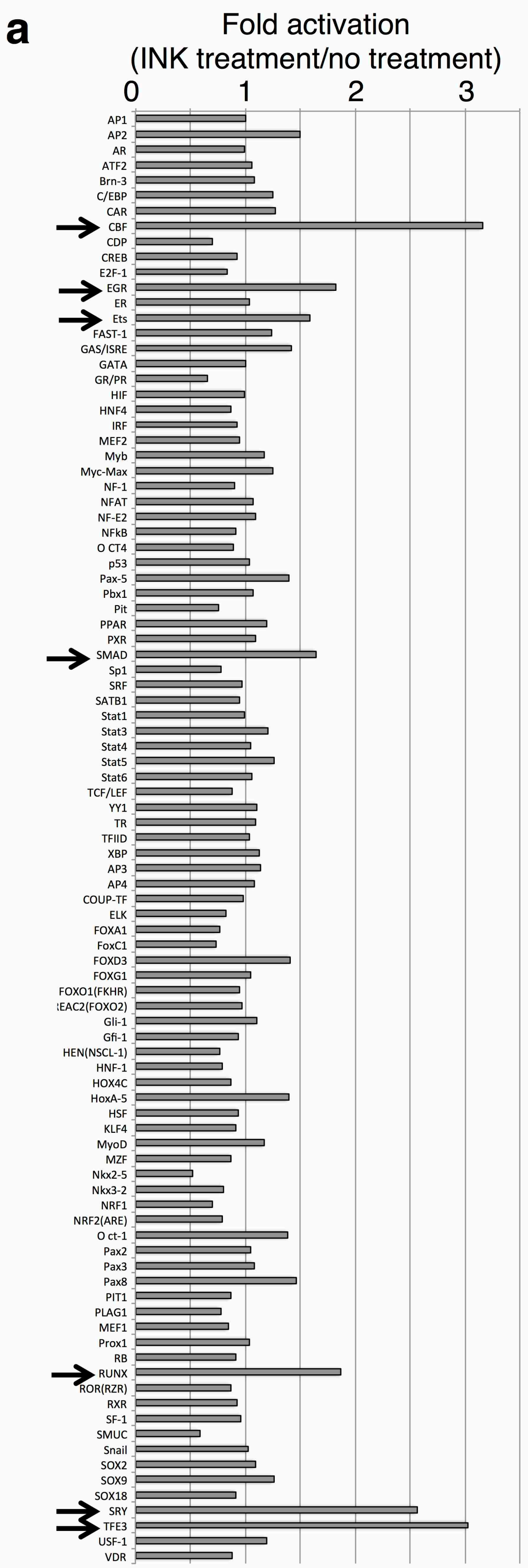

Supplementary Figure 7. Transcription factors activated upon mTOR inhibitor treatment

b

Name	Score	Relative score	Strand	predicted site sequence
TFE3	10.139	0.920	-1	ACCACGTGCA
RUNX2	7.874	0.871	-1	TAACCGCCT
TFE3	7.683	0.873	1	TGCACGTGGT
RUNX1	6.061	0.816	-1	CGTTGAGGTCG
EGR1	6.317	0.848	1	GCTCCTCCCGCTCG
RUNX2	5.557	0.829	1	CGACCTCAA
EGR2	5.577	0.823	-1	CCGACCACGTG
ETS1	5.486	0.827	-1	GCTGGAAATG
RUNX3	5.291	0.818	-1	TAACCGCCTG
RUNX3	4.775	0.809	1	CGACCTCAAC
EGR1	2.594	0.808	-1	CCGACGGTCCCGCC
EGR1	2.563	0.808	-1	GTCACGCCCAGGGC
EGR1	2.146	0.803	-1	CAGCCGACCACGTG
EGR1	1.863	0.800	1	CCGCCTCAGACTCA

Supplementary Figure 7. Transcription factors activated upon mTOR inhibitor treatment

(**a**) MiaPaCa-2 cells were treated with INK (100 nM) for 1 hr. Activation of each transcription factors was measured using the TF Activation Profiling Plate Array II (for 96 TFs). Arrows show the transcription factors activated by the treatment of INK.

(**b**) Possible transcription factor binding sites in *EIF4EBP3* promoter (-163 to +59) predicted by Jaspar (http://jaspar.genereg.net).

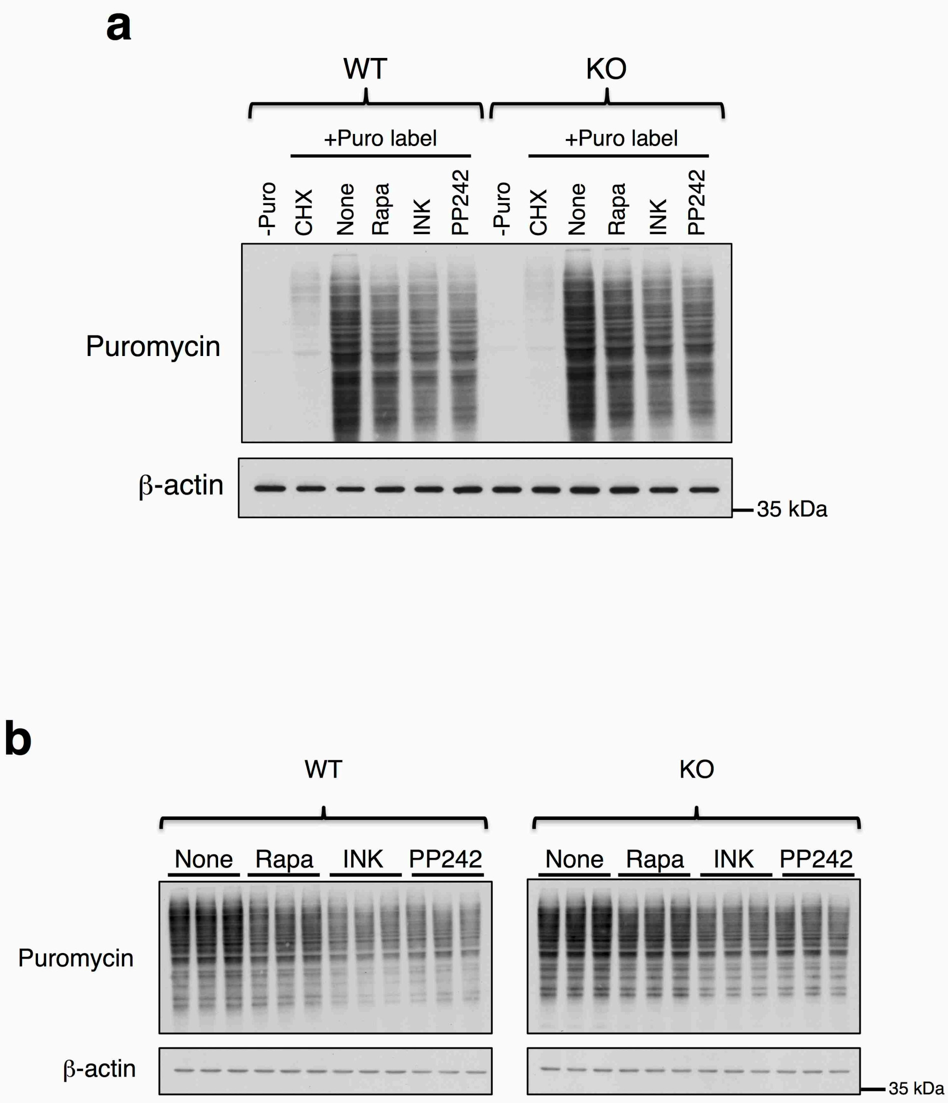

Supplementary Figure 8. Effects of 4E-BP3 on protein synthesis

(**a, b**)Protein synthesis was monitored using non-radioactive method (SUnSET). 4E-BP3 WT or KO MiaPaCa-2 cells were pretreated with rapamycin (100 nM), INK1341 (100 nM) or PP242 (1 μM) for 24 h. Then, cells were labelled with 2 μg ml^{-1} puromycin for 15 min in the presence or absence of cycloheximide (100 μg ml^{-1}). Extracts were processed for western blotting using the indicated antibodies.

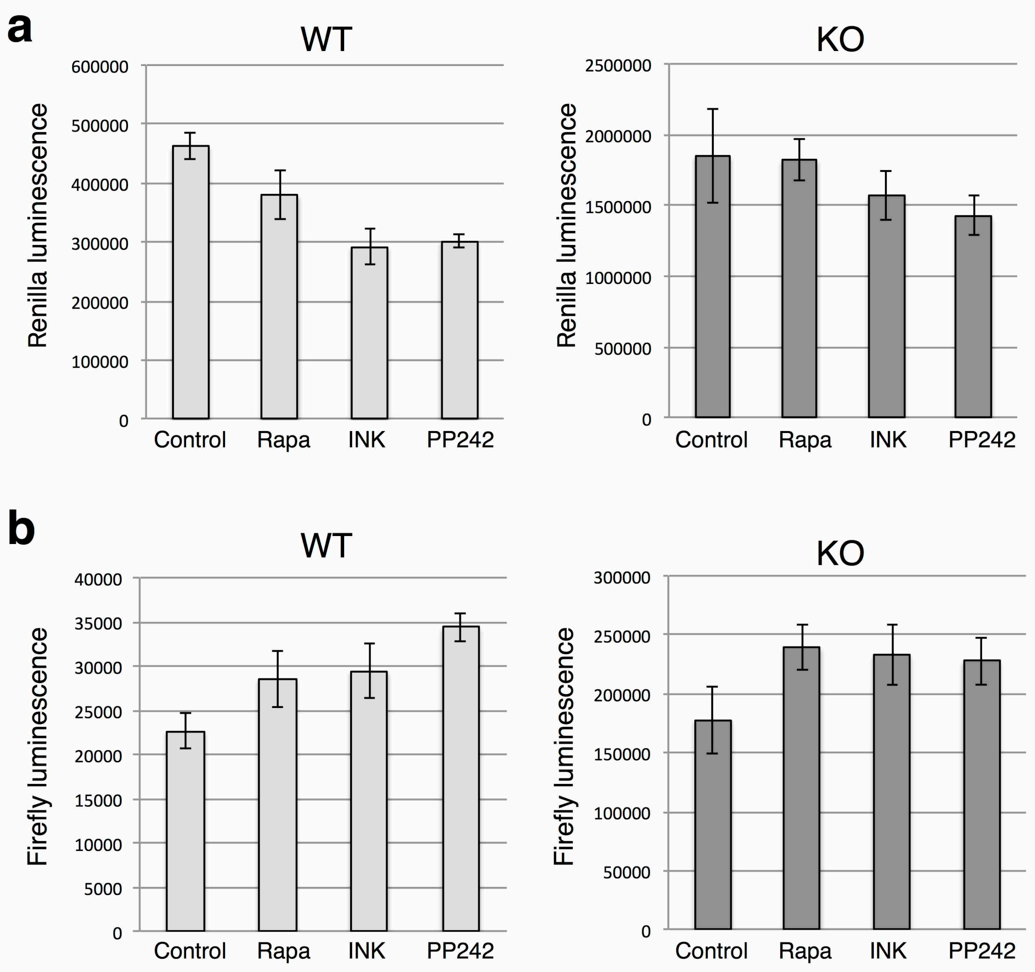

Supplementary Figure 9. Effects of 4E-BP3 on bicistronic reporter activity

(**a, b**) 4E-BP3 WT or KO MiaPaCa-2 cells were transfected with the bicistronic reporter plasmid construct for 24 hr and then treated with mTOR inhibitors for 12 hr. Each luciferase activity was measured using dual luciferase assay kit (Promega). Error bars indicate ± SD (n=3). Renilla luminescence (a), Firefly luminescence (b.)

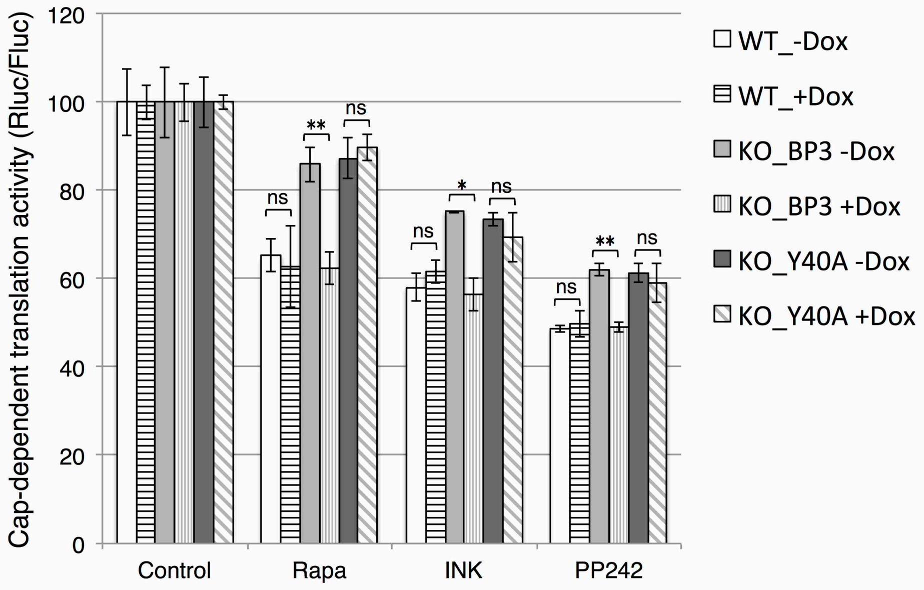

Supplementary Figure 10. Dox-induced 4E-BP3 in KO cells augments cap-dependent translation repression upon mTOR inhibitor treatment

Cells expressing doxycycline-inducible 4E-BP3 or the Y40A mutant were generated from 4E-BP3 KO MiaPaCa-2 cells. Cells were transfected with the bicistronic reporter plasmid construct for 24 hr and then treated with mTOR inhibitors in the presence or absence of doxycycline for 12 hr. Each luciferase activity was measured using dual luciferase assay kit (Promega). Error bars indicate ± SD (n=3). *$p<0.05$. **$p<0.01$. The statistical difference between each group was determined using Student t-test.

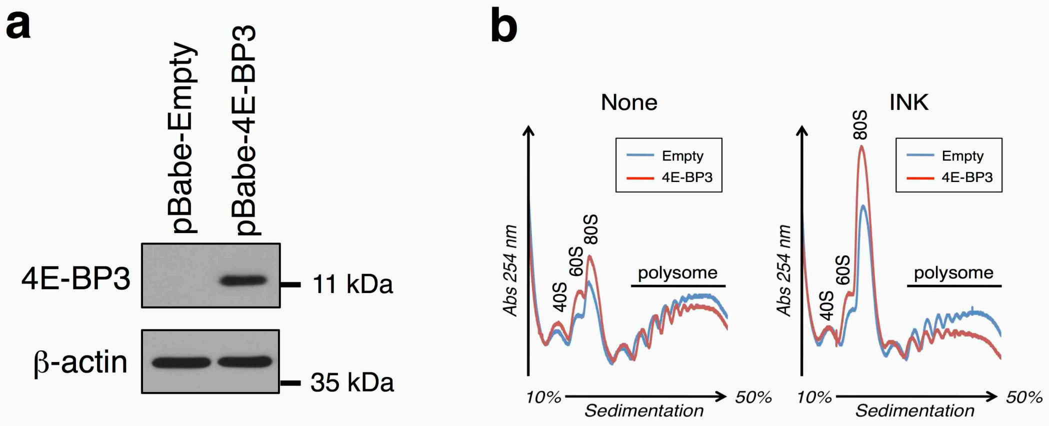

Supplementary Figure 11. 4E-BP3 overexpression decreases polysome fractions upon mTOR inhibitor treatment

(**a**) 293T cells were transduced with pBabe-empty, or pBabe-4E-BP3 retroviral vector and stable cell lines were established by puromycin selection.

(**b**) Polysome profiling in 4E-BP3-overexpressed 293T cells treated with 30 nM of INK1341 for 1 hr.

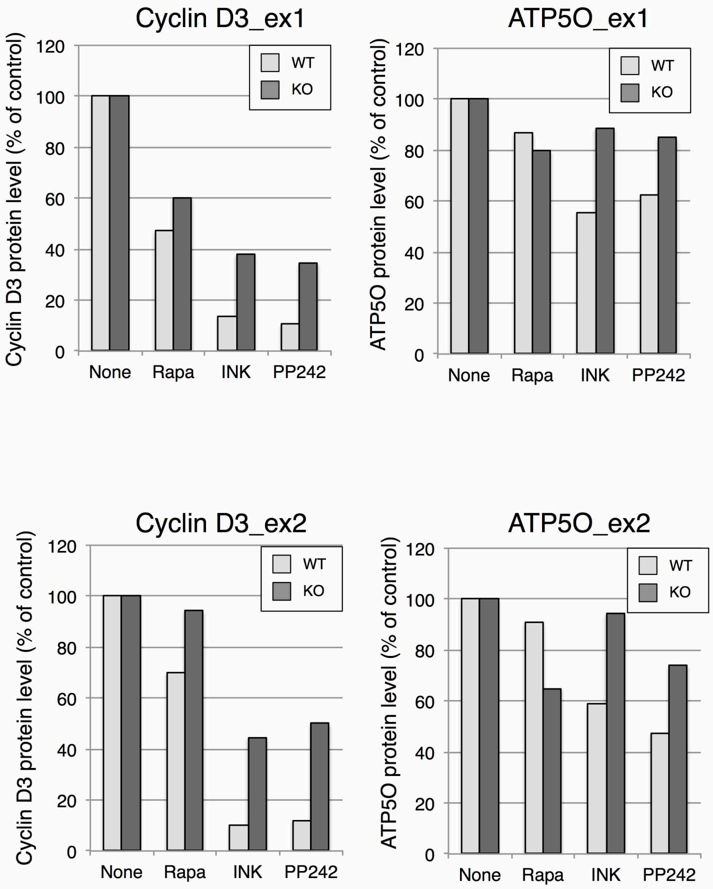

Supplementary Figure 12. Quantification of cyclin D3 and ATP5O protein level

Cyclin D3(left) and ATP5O(right) protein expressions in figure 3h were quantified by densitometry analysis using ImageJ and normalized by β-actin level. The results of two independent experiments (ex1 or ex2) were shown.

a

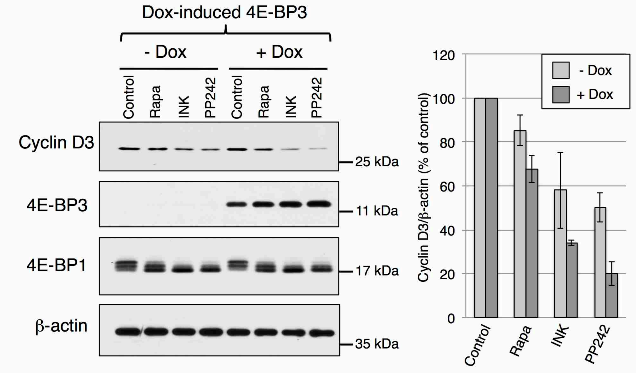

b

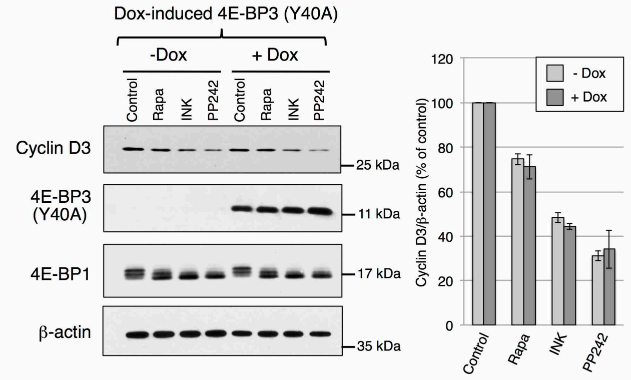

Supplementary Figure 13. Dox-induced 4E-BP3 in KO cells results in repressed cyclin D3 protein expression upon mTOR inhibitor treatment

(**a, b**) Cells expressing doxycycline-inducible 4E-BP3 (a) or the Y40A mutant (b) were treated with mTOR inhibitors in the presence or absence of doxycycline for 12 hr. Cyclin D3 protein expression was quantified by densitometry analysis using ImageJ (right panels) and normalized to β-actin. Error bars indicate ± SD (n=3).

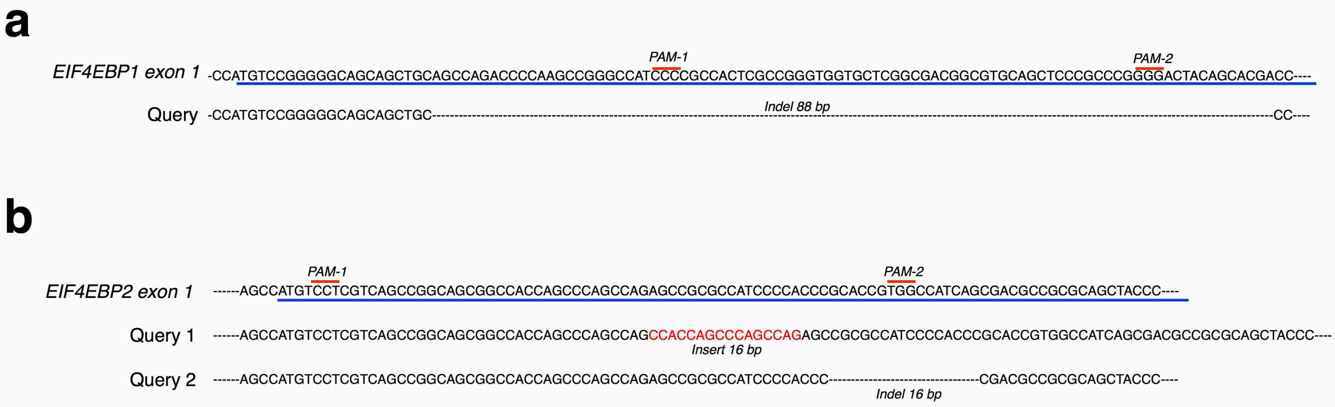

Supplementary Figure 14. Generation of human *EIF4EBP1,2,3* triple knockout (TKO) cells

(**a, b**) Indel or insertion mutation in *EIF4EBP1* and *EIF4EBP2* exon 1 was generated by the CRISPR-Cas9 nickase system. Protospacer adjacent motif (PAM) is highlighted in red. Protein coding region is underlined in blue.

```
                                                                PAM-1
EIF4EBP1 exon1   GCACAGGAGACCATGTCCGGGGGCAGCAGCTGCAGCCAGACCCCAAGCCGGGCCATCCCC
         Query   GCACAGGAGACCATGTCCGGGGGCAGCAGCTGCAGCCAGACCCCAAGCCGGGCCATCCCC
                 ************************************************************

                                                       PAM-2
EIF4EBP1 exon1   GCCACTCGCCGGGTGGTGCTCGGCGACGGCGTGCAGCTCCCGCCCGGGGACTACAGCACG
         Query   GCCACTCGCCG-------------------------------------------------
                 ***********                                  Indel 70 nt

EIF4EBP1 exon1   ACCCCCGGCGGCACGCTCTTCAGCACCACCCCGGGAG
         Query   ---------------------AGCACCACCCCGGGAG
                                      ****************
```

Supplementary Figure 15. Generation of human *EIF4EBP1* knockout cells

Indel mutation in *EIF4EBP1* exon 1 was generated by the Crispr-Cas9 nickase system. Protospacer adjacent motif (PAM) is highlighted in red. Protein coding region is underlined in blue.

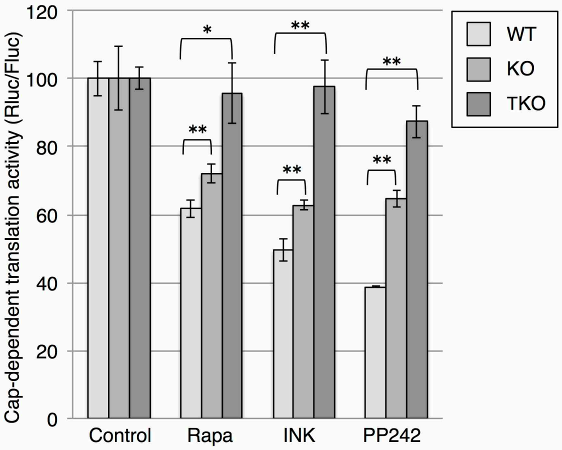

Supplementary Figure 16. Cap-dependent translation activity is sustained at higher level in TKO cells during mTOR inhibition

4E-BP3 WT, KO or 4E-BP1,2,3 TKO MiaPaCa-2 cells were transfected with the bicistronic reporter plasmid construct for 24 hr and then treated with mTOR inhibitors for 12 hr. Luciferase activity was measured using the dual luciferase assay kit (Promega). Error bars indicate ± SD (n=3). *p<0.05. **p<0.01. The statistical difference between each group was determined using Student t-test.

a

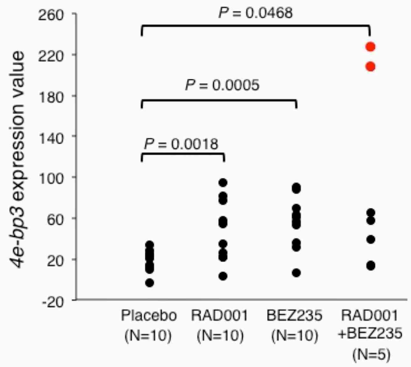

b

DEN-induced liver tumors				
Placebo (N=10)	RAD001 (N=10)	BEZ235 (N=10)	RAD+BEZ (N=7)	**Gene**
1.0	2.5 (0.0118)	3.0 (0.0005)	2.0 (0.0468)	*4e-bp3*
1.0	1.0 (0.9782)	0.9 (0.2956)	1.0 (0.8873)	*4e-bp1*
1.0	0.9 (0.5103)	0.9 (0.4304)	1.0 (0.7619)	*4e-bp2*
1.0	0.9 (0.0712)	0.9 (0.0966)	0.8 (0.0323)	*Actb*
1.0	1.2 (0.2689)	1.0 (0.9846)	1.2 (0.0890)	*Gapdh*

Supplementary Figure 17. Single treatment of mTOR-targeting drug is sufficient to induce *4e-bp3* mRNA in tumors

(**a, b**) The dataset was obtained from the Gene Expression Omnibus (GEO) database, www.ncbi.nlm.nih.gov/geo (accession no. GSE37129) (Thomas et al., 2012). RAD001 or BEZ235 administration induces *4e-bp3*, but not *4e-bp1* and *2*, in vivo. Male C57BL/6 mice were injected with DEN at 2 weeks. After 44 weeks, mice were treated daily with placebo, RAD001, BEZ235 alone or combination. (a, b). Relative change in gene expression was determined by dividing the average of expression value in drug-treated samples by the average of expression values in placebo (b). For samples treated with RAD001 and BEZ235 combination, two samples were considered as outlier expression value (red circle) and removed from calculation of relative change in *4e-bp3* expression (N=5). The statistical difference between each group was determined using Student t-test.

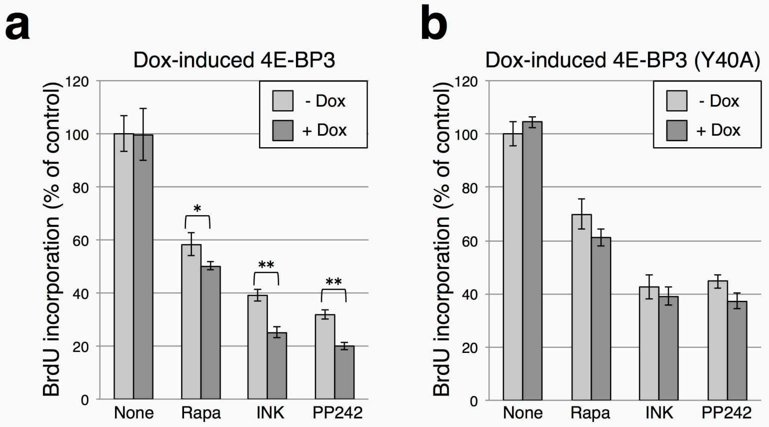

Supplementary Figure 18. Dox-induced 4E-BP3 expression resensitizes KO cells to mTOR inhibitors

(**a, b**) MiaPaCa-2 KO cells expressing doxycycline-inducible 4E-BP3 (A) or the Y40A mutant (B) were treated with mTOR inhibitors in the presence or absence of doxycycline for 72 hr. Cell proliferation was measured by BrdU incorporation. Error bars indicate ± SD (n=3). *p<0.05.**p<0.01. The statistical difference between each group was determined using Student t-test.

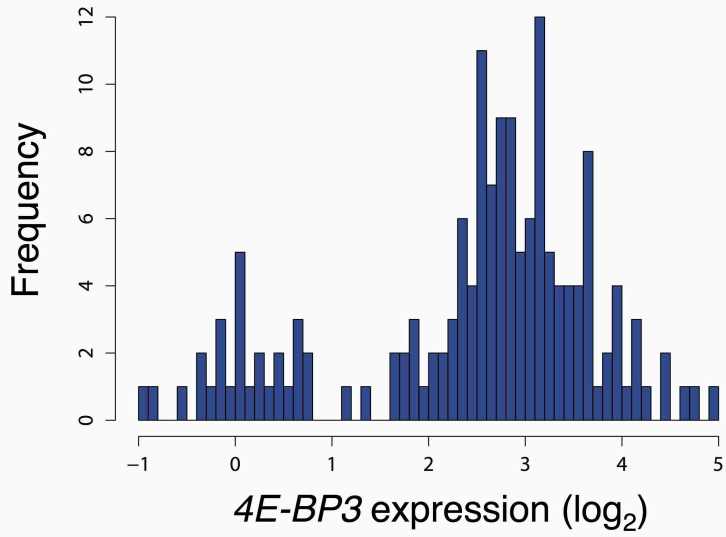

Supplementary Figure 19. Bimodal expression of *4E-BP3* in human breast cancer patients

The distribution of *4E-BP3* expression values suggested two distinct populations. Patients with log2 expression values < 1 were categorized as having low expression and patients with values ≥ 1 were categorized as having high expression.

4E−BP3 level	High (n=128)	Low (n=27)
Age average	67.2	67.3
Adjuvant therapy	Tam 21.1%, Xray+Tam 73.4%, Xray +Tam_LHRH 5.5%	Tam 22.2%, Xray+Tam 77.8%
ER-neative	5.5%	3.7%
PR-negative	16.4%	29.6%
pT (TNM)	T1 37.5%, T2 56.3%, T3 1.6%, T4 1.6%	T1 37.0%, T2 59.3%, T3 3.7%
pN (TNM)	N0 59.4%, N1 38.3%	N0 7.4%, N1 85.2%
Distant metastasis	23.4%	63.0%

Supplementary Figure 20. Increased metastasis in patients with low 4E-BP3 expression

155 breast cancer patients were classified with 4E-BP3 expression level. These patients were either ER-positive or PR-positive and all patients in this cohort were treated with tamoxifen (Tam) or in combination with radiation (X-ray) and hormone agonist (LHRH).

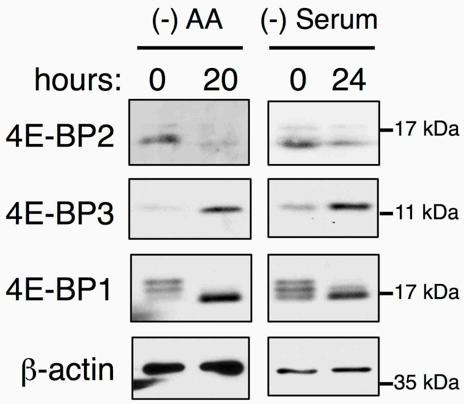

Supplementary Figure 21. Nutrient starvation reduces 4E-BP2 expression while it induces 4E-BP3 expression

MiaPaCa-2 cells were incubated without amino acids for 20 hr or without serum for 24 hr. Expression of the indicated proteins was determined by immunoblotting.

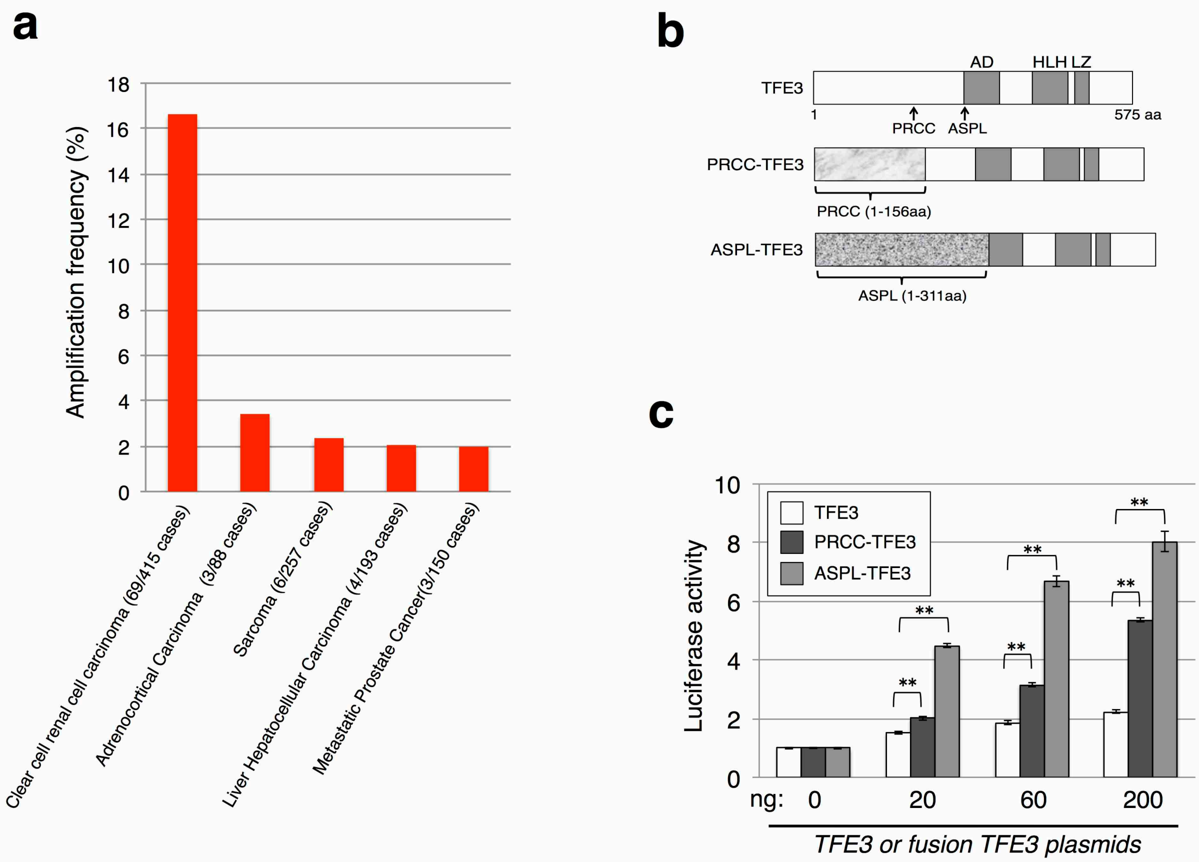

Supplementary Figure 22. Upregulation of 4E-BP3 in a subset of renal cell carcinoma

(**a**) Amplification of *EIF4EBP3* locus in several human cancers. The data were obtained from the cBioPortal (http://www.cbioportal.org).

(**b**) Schematic structures of TFE3, PRCC-TFE3 and ASPL-TFE3 chimeric proteins.

AD: transcription activation domain, HLH: helix-loop-helix, LZ: leucine zipper, Break points for fusion protein are shown by arrows.

(**c**) 293T cells were transiently transfected with the indicated amounts of TFE3, PRCC-TFE3 or ASPL-TFE3 plasmid together with reporter plasmids (4E-BP3pro and pRL-TK). After 48 hr, luciferase activity was measured using dual luciferase assay kit. PRCC-TFE3 or ASPL-TFE3 fusion plasmid was obtained by PCR amplification of cDNAs prepared from UOK146 (which is expressing PRCC-TFE3) or FU-UR1 (which is expressing ASPL-TFE3) cells. Error bars indicate ± SD (n=3). **p<0.01. The statistical difference between each group was determined using Student t-test.

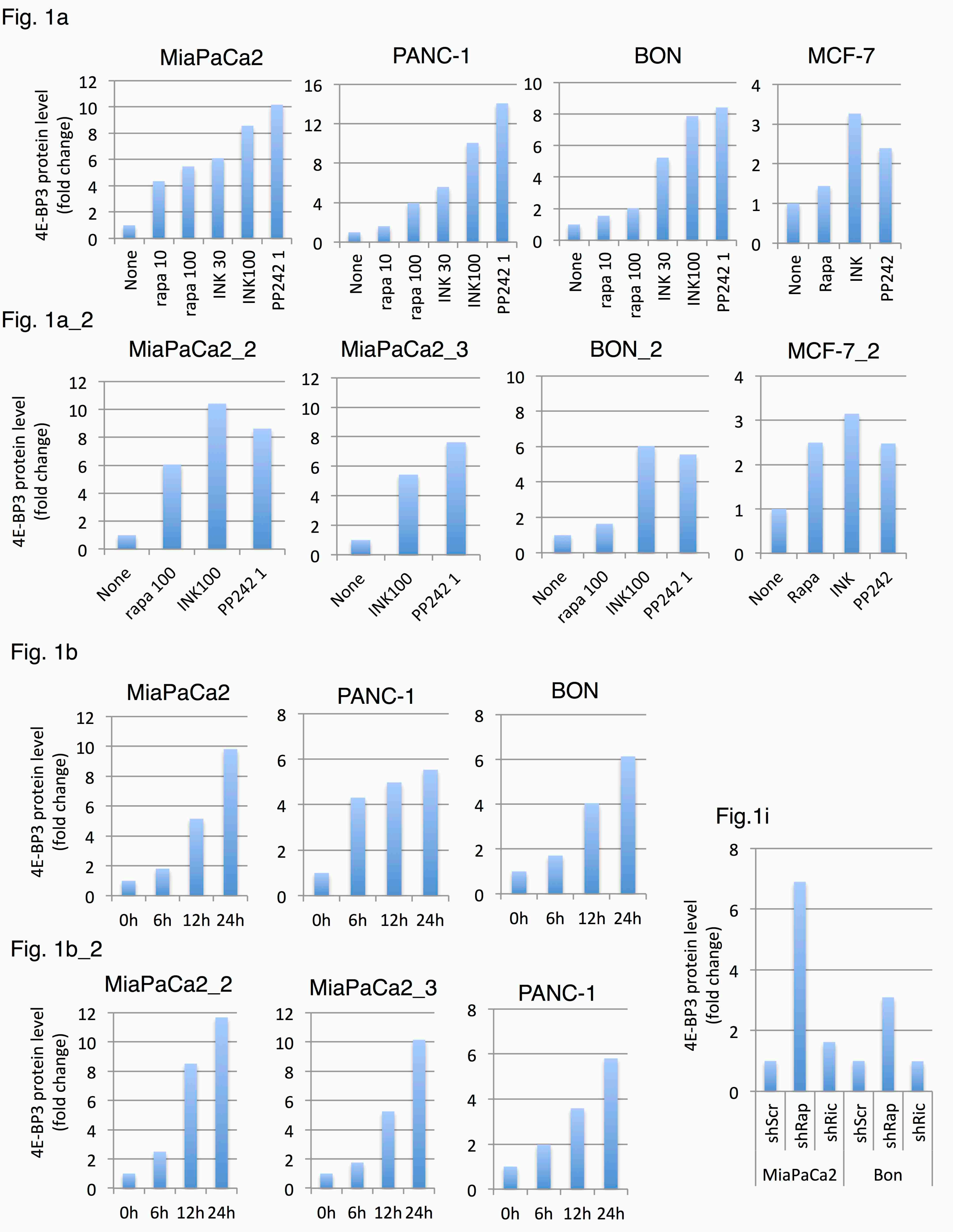

Supplementary Figure 23. Densitometry analysis of 4E-BP3 proteins

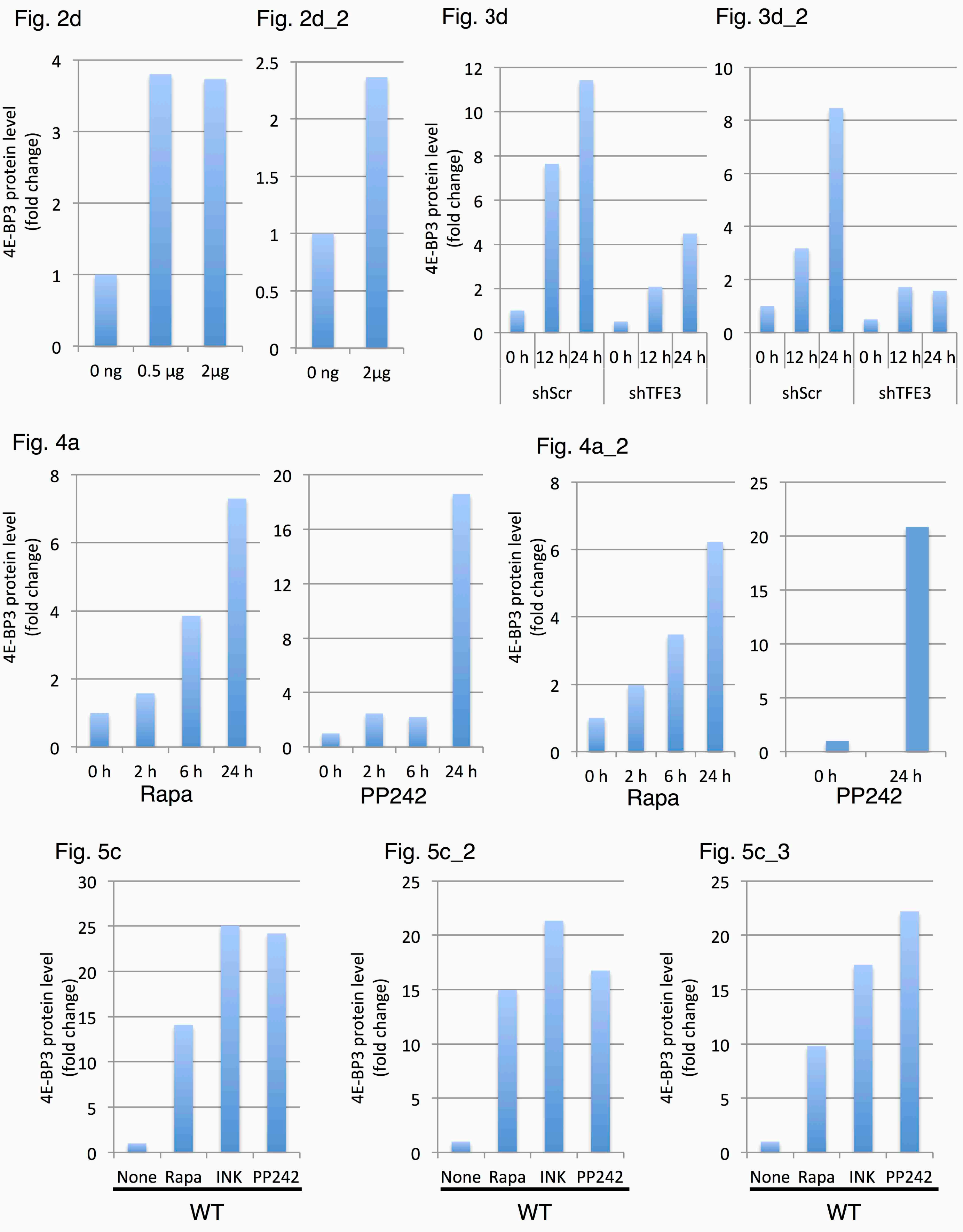

Supplementary Figure 23. Densitometry analysis of 4E-BP3 proteins

Fig. 6d

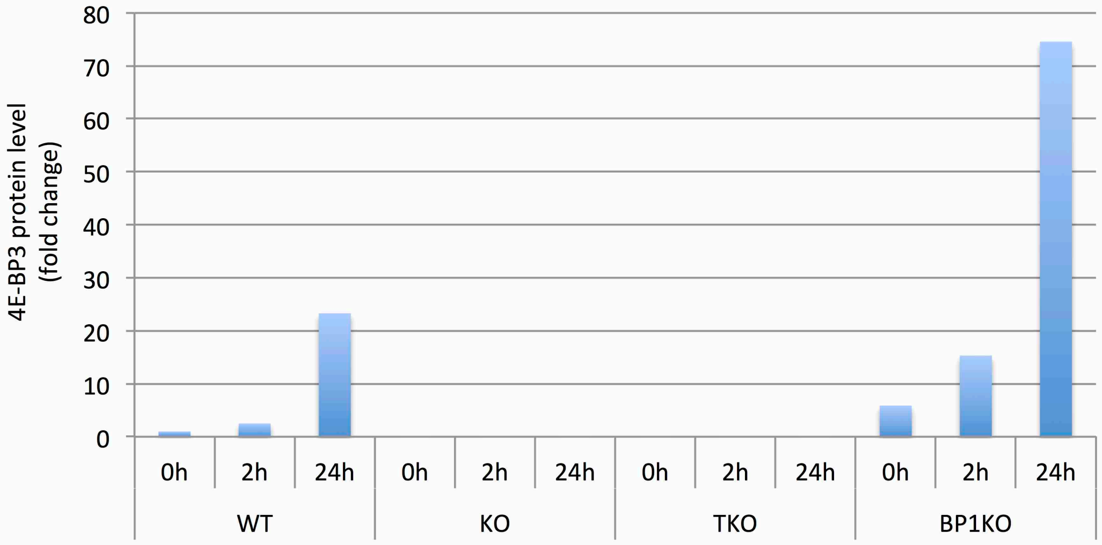

Fig. 6d_2

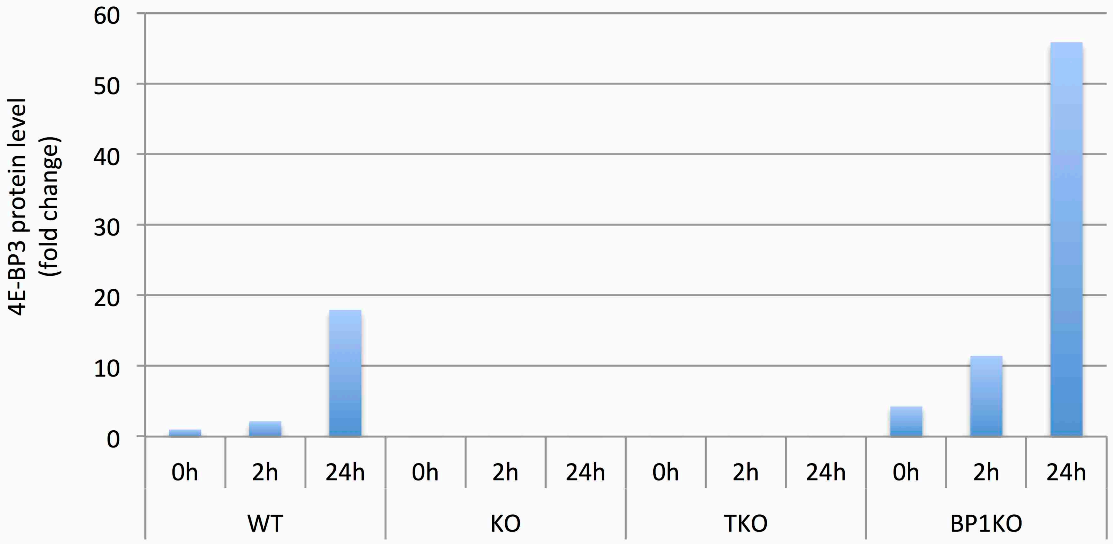

Supplementary Figure 23. Densitometry analysis of 4E-BP3 proteins

RT-PCR	
human 4E-BP1_F	ATGGAGTGTCGGAACTCACC
human 4E-BP1_R	TGGAGGCACAAGGAGGTATC
human 4E-BP2_F	TTCCAGCTCACTGGGACTCT
human 4E-BP2_R	CCCATGTGCATGTAGTCCAG
human 4E-BP3_F	CGGTTACTTTGCTGCTCCTC
human 4E-BP3_R	GTGCGTCATCGGGTATCTCT
human beta-actin_F	CATCACCATTGGCAATGAGC
human beta-actin_R	GCCATGCCAATCTCATCTTG
human GAPDH_F	CTTTGGTATCGTGGAAGGAC
human GAPDH_R	CAGCAGTGAGGGTCTCTCTC
human TFE3_F	GTCCTGGCACAGAAGAGGAG
human TFE3_R	GCTCAGAGCCCTCCTAGGTT
human TFEB_F	ACCCTGAGAGGGAGTTGGAT
human TFEB_R	TTGATGCGGTCATTGATGTT
mouse 4e-bp3_F	GAAAGTTCCTGCTGGAGTGC
mouse 4e-bp3_R	CCTGGTCTAGCTGCCTCAAC
mouse beta-actin_F	TGTTACCAACTGGGACGACA
mouse beta-actin_R	TCTCAGCTGTGGTGGTGAAG

qPCR	
human 4E-BP1_F	TCACCTGTGACCAAAACACC
human 4E-BP1_R	CTTGGTAGTGCTCCACACGA
human 4E-BP2_F	AAGAGCCCAGAGGACACTCA
human 4E-BP2_R	TCATGGGAGACAAAGGGAAG
human 4E-BP3_F	CGGTTACTTTGCTGCTCCTC
human 4E-BP3_R	CGGTCGTAGATGATCCTGGT
human beta-actin_F	CTGTGGCATCCACGAAACTA
human beta-actin_R	AGTACTTGCGCTCAGGAGGA
human GAPDH_F	CAGCCTCAAGATCATCAGCA
human GAPDH_R	GTCTTCTGGGTGGCAGTGAT
human SQSTM1_F	CTTCTCTTGAGGCCTGTGCT
human SQSTM1_R	GAGCTATGGAGTCCCATCCA
human VPS8_F	TCTCTACCGAGGAAGCTCCA
human VPS8_R	ACAGGTGGAGGAATGAGCTG
human VPS11_F	AGAGGAGACCACCCGTATCC
human VPS11_R	CTCAAAGCAGTGTTGGTGGA
human ATP5G1_F	TGTGTCTGCCTCCTTCTTGA
human_ATP5G1_R	CTTGGCTGCTGTGTCAATGT
human CCND3_F	CTGGATCGCTACCTGTCTTG
human CCND3_R	TCCCACTTGAGCTTCCCTAG
human ATP5O_F	CTCTCTTCCCACTCGGGTTT
human ATP5O_R	TGACCACAGAGGTACTGAAGCA

ChIP assay	
Primer 1 F	CTTAGCCTCCCAAAGTGCTG
Primer 1 R	GCCAAAGTCACACATCTTGC
Primer 2 F	GGCTGGCTTCCTAGCAGATA
Primer 2 R	GGCGTTGAGGTCGAGGAG
Primer 3 F	CCAGCTGGCCTCATCTAATC
Primer 3 R	TACTCCATTCCCCAGTTCCA

Supplementary Figure 24. Primer information

Fig 1_a

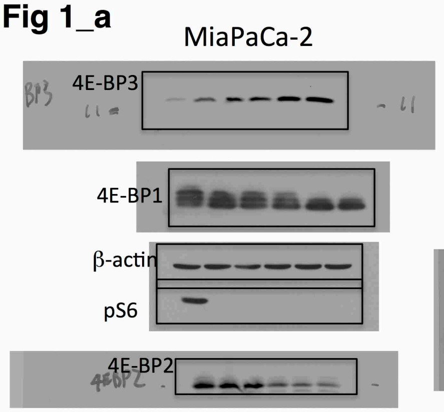

Fig 1_a

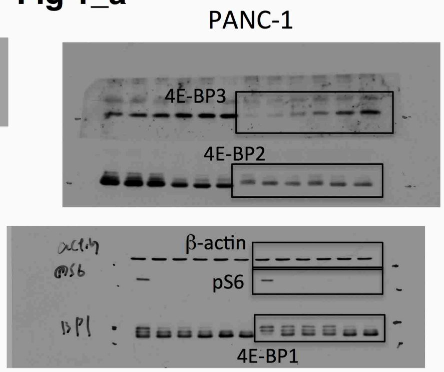

Fig 1_a

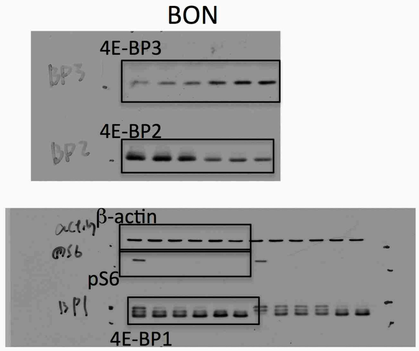

Fig 1_a

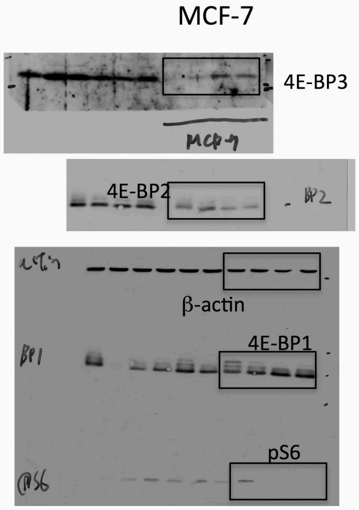

Fig 1_b

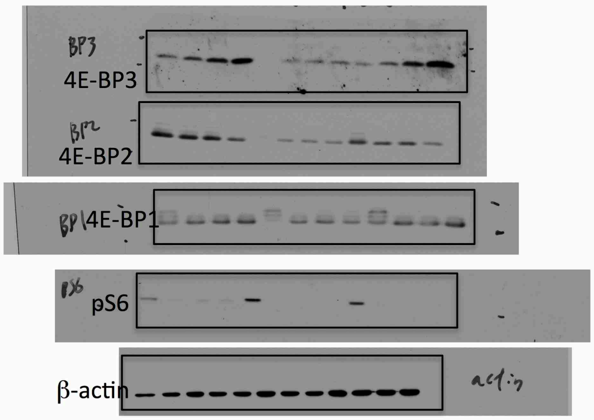

Supplementary Figure 25 Uncropped scanned images

Fig 1_c

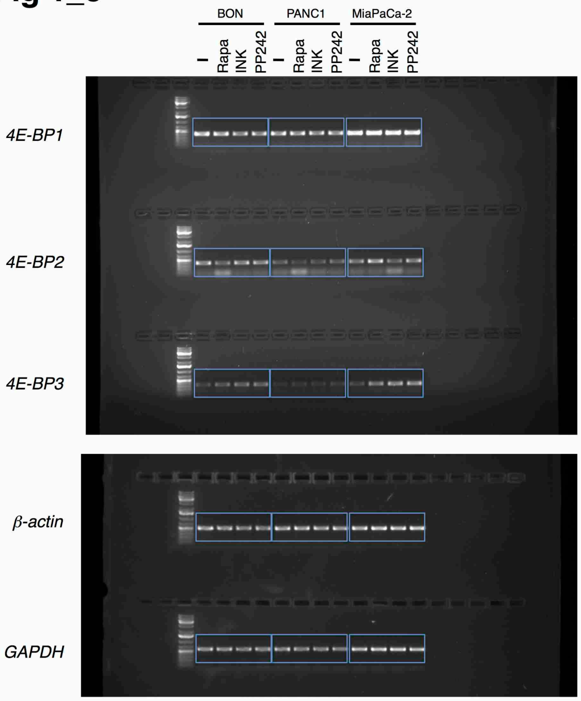

Fig 1_e

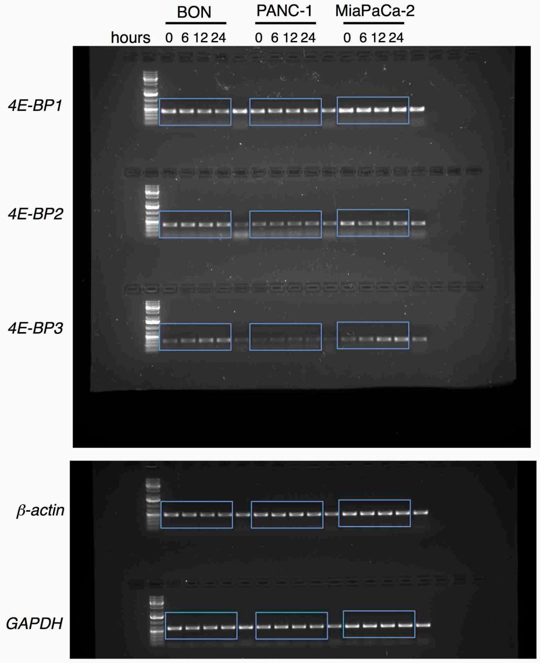

Fig 1_g

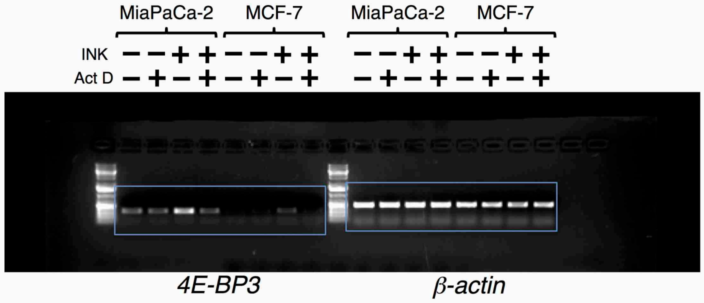

Fig 1_i

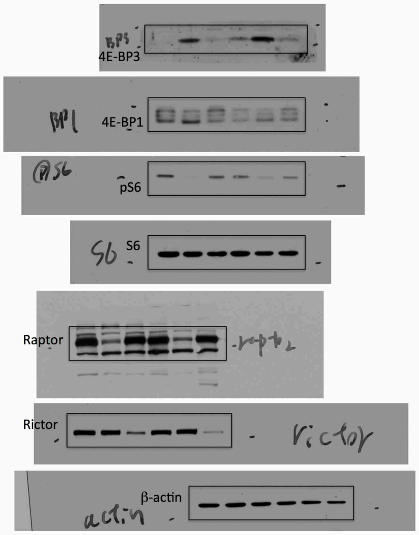

Supplementary Figure 25 Uncropped scanned images

Fig 2_c

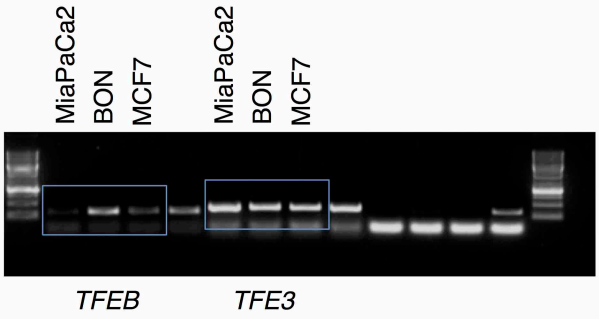

Fig 2_c

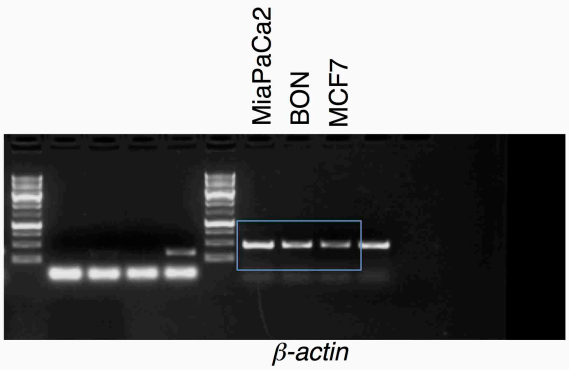

Fig 2_d

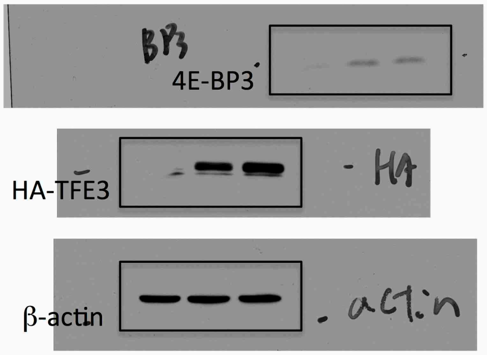

Fig 3_b

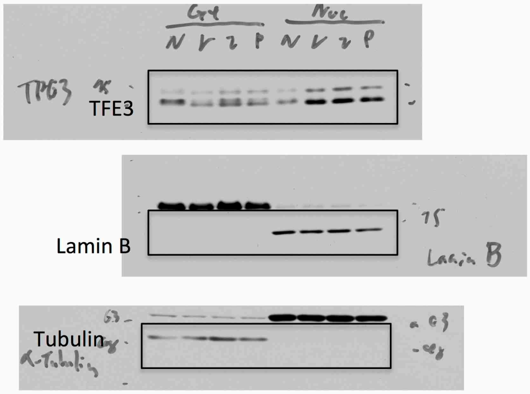

Fig 3_c

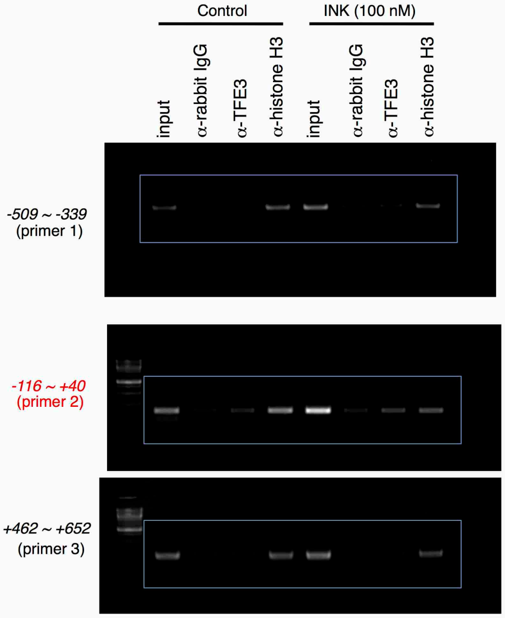

Fig 3_d

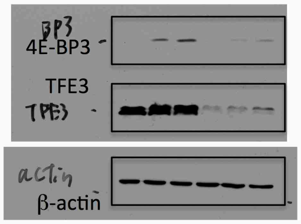

Supplementary Figure 25 Uncropped scanned images

Fig 4_a

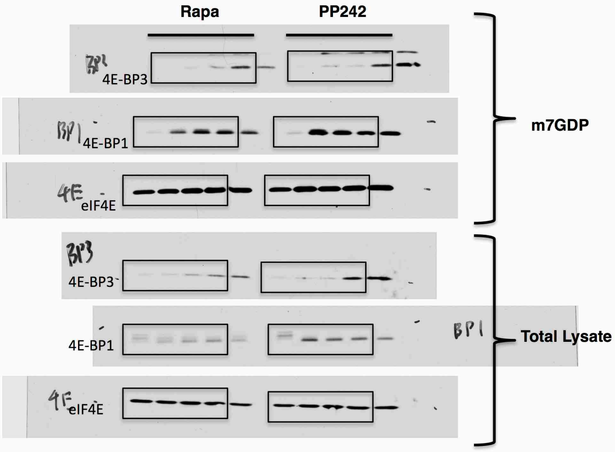

Fig 5_d

Fig 5_c

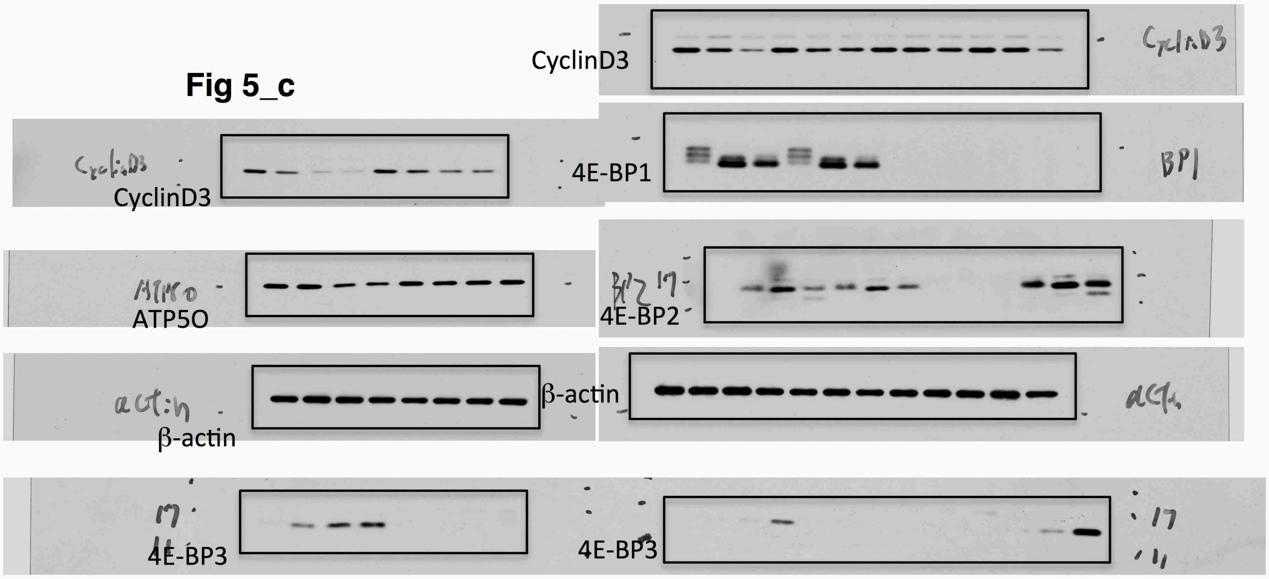

Supplementary Figure 25 Uncropped scanned images

Fig S2_a

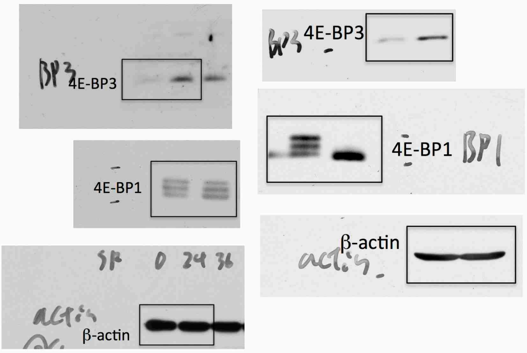

Fig S2_b

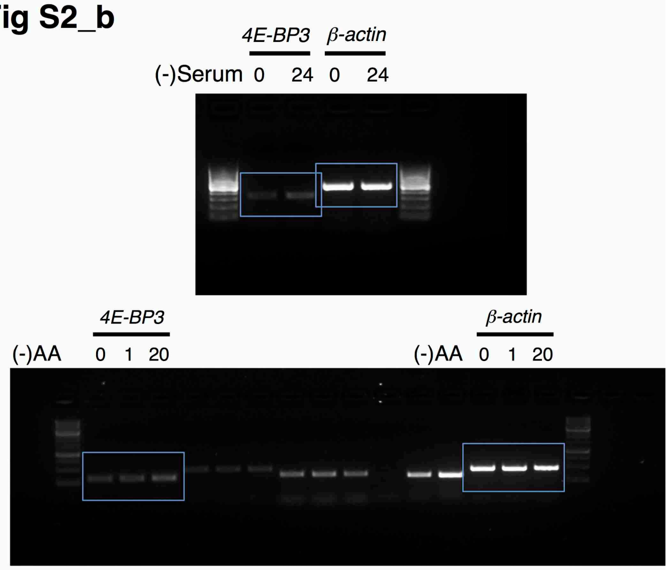

Fig S2_c

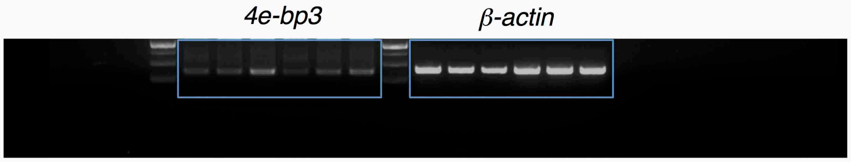

Fig S3_a

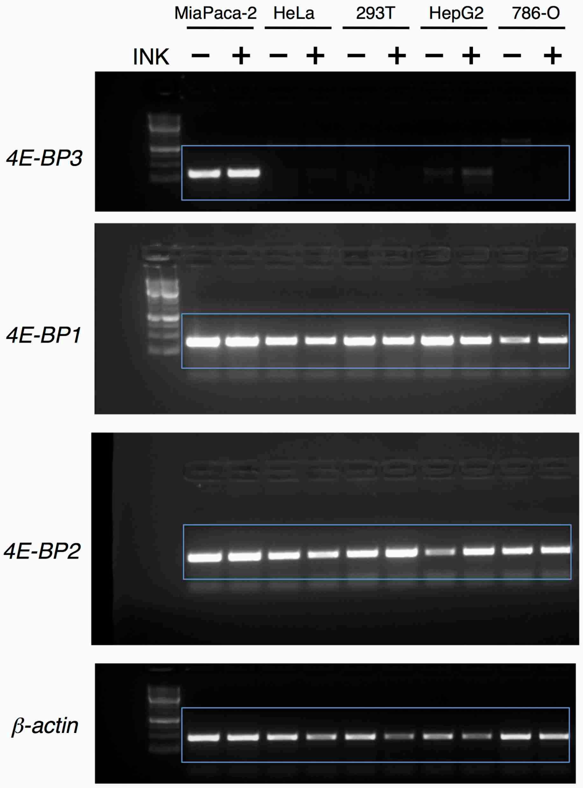

Fig S3_b

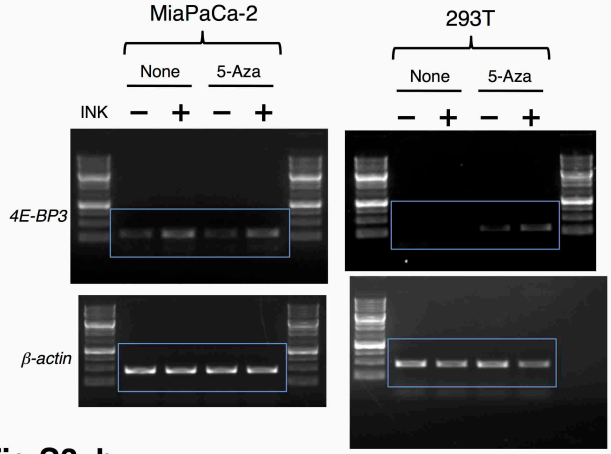

Fig S3_b

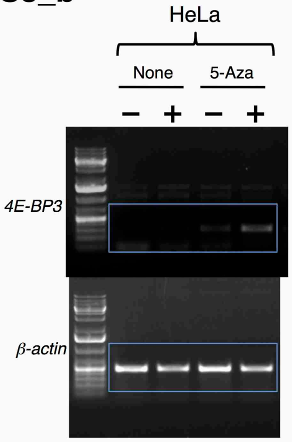

Supplementary Figure 25 Uncropped scanned images

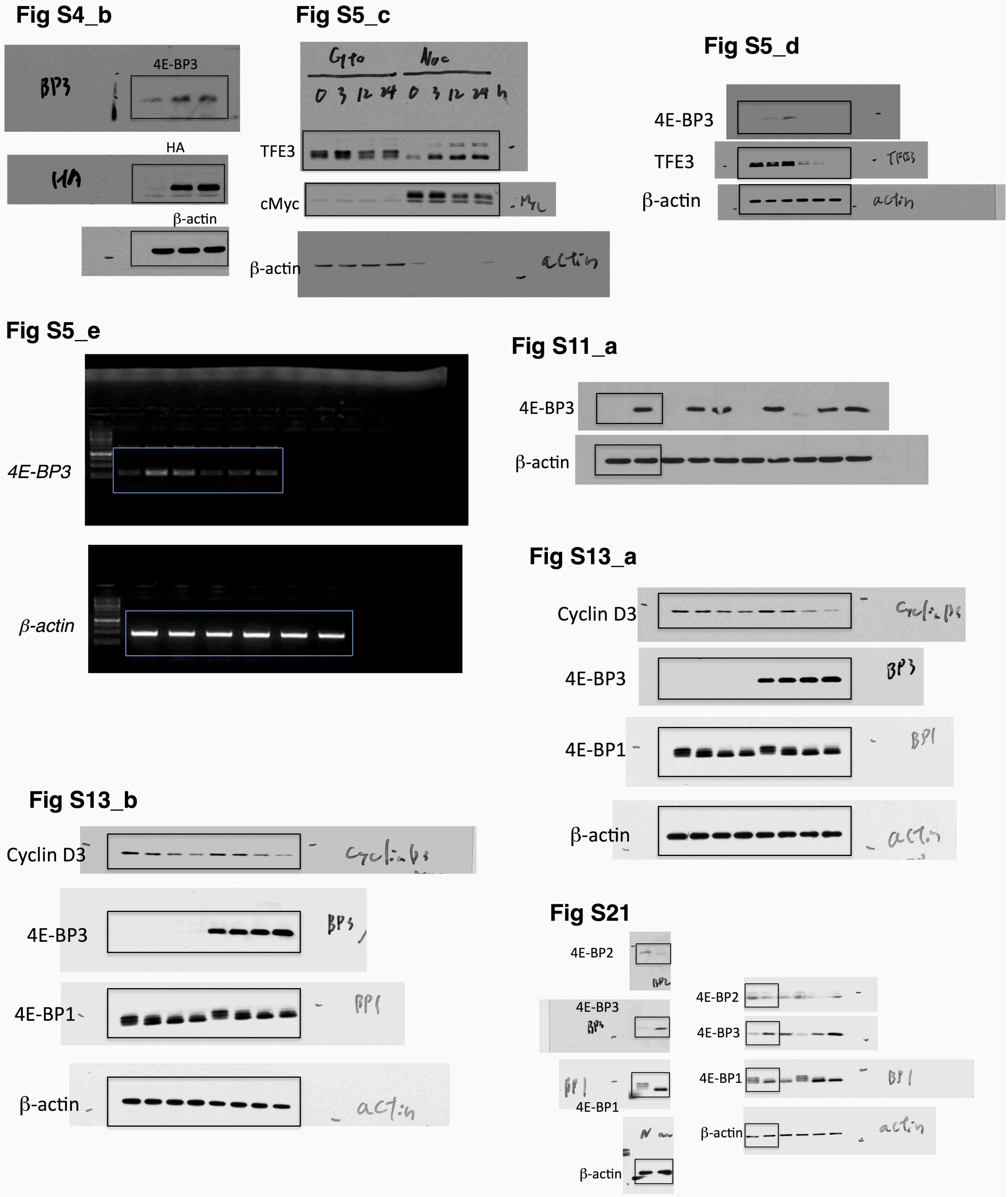

Supplementary Figure 25 Uncropped scanned images
